# Competition between Jagged-Notch and Endothelin1 Signaling Selectively Restricts Cartilage Formation in the Zebrafish Upper Face

**DOI:** 10.1371/journal.pgen.1005967

**Published:** 2016-04-08

**Authors:** Lindsey Barske, Amjad Askary, Elizabeth Zuniga, Bartosz Balczerski, Paul Bump, James T. Nichols, J. Gage Crump

**Affiliations:** 1 Eli and Edythe Broad CIRM Center for Regenerative Medicine and Stem Cell Research, University of Southern California Keck School of Medicine, Los Angeles, California, United States of America; 2 Institute of Neuroscience, University of Oregon, Eugene, Oregon, United States of America; University of Pennsylvania School of Medicine, UNITED STATES

## Abstract

The intricate shaping of the facial skeleton is essential for function of the vertebrate jaw and middle ear. While much has been learned about the signaling pathways and transcription factors that control facial patterning, the downstream cellular mechanisms dictating skeletal shapes have remained unclear. Here we present genetic evidence in zebrafish that three major signaling pathways − Jagged-Notch, Endothelin1 (Edn1), and Bmp − regulate the pattern of facial cartilage and bone formation by controlling the timing of cartilage differentiation along the dorsoventral axis of the pharyngeal arches. A genomic analysis of purified facial skeletal precursors in mutant and overexpression embryos revealed a core set of differentiation genes that were commonly repressed by Jagged-Notch and induced by Edn1. Further analysis of the pre-cartilage condensation gene *barx1*, as well as *in vivo* imaging of cartilage differentiation, revealed that cartilage forms first in regions of high Edn1 and low Jagged-Notch activity. Consistent with a role of Jagged-Notch signaling in restricting cartilage differentiation, loss of Notch pathway components resulted in expanded *barx1* expression in the dorsal arches, with mutation of *barx1* rescuing some aspects of dorsal skeletal patterning in *jag1b* mutants. We also identified *prrx1a* and *prrx1b* as negative Edn1 and positive Bmp targets that function in parallel to Jagged-Notch signaling to restrict the formation of dorsal *barx1*+ pre-cartilage condensations. Simultaneous loss of *jag1b* and *prrx1a/b* better rescued lower facial defects of *edn1* mutants than loss of either pathway alone, showing that combined overactivation of Jagged-Notch and Bmp/Prrx1 pathways contribute to the absence of cartilage differentiation in the *edn1* mutant lower face. These findings support a model in which Notch-mediated restriction of cartilage differentiation, particularly in the second pharyngeal arch, helps to establish a distinct skeletal pattern in the upper face.

## Author Summary

The exquisite functions of the vertebrate face require the precise formation of its underlying bones. Remarkably, many of the genes required to shape the facial skeleton are the same from fish to man. In this study, we use the powerful zebrafish system to understand how the skeletal components of the face acquire different shapes during development. To do so, we analyze a series of mutants that disrupt patterning of the facial skeleton, and then assess how the genes affected in these mutants control cell fate in skeletal progenitor cells. From these genetic studies, we found that several pathways converge to control when and where progenitor cells commit to a cartilage fate, thus controlling the size and shape of cartilage templates for the later-arising bones. Our work thus reveals how regulating the timing of when progenitor cells make skeleton helps to shape the bones of the zebrafish face. As mutations in many of the genes studied are implicated in human craniofacial defects, differences in the timing of progenitor cell differentiation may also explain the wonderful diversity of human faces.

## Introduction

Morphogenesis of the facial skeleton in zebrafish is tightly linked with the early differentiation of pharyngeal arch neural crest-derived cells (NCCs) into cartilage. Shortly after migration into the pharyngeal arches, NCCs form a series of pre-cartilage condensations that prefigure the distinct shapes of the later cartilage-replacement bones. As near-isometric growth of these cartilages during the later larval period largely preserves these initial shapes [1], early patterning, not later growth, is the major determinant of facial skeletal shaping. Identifying the local signals that sculpt and arrange early condensations in specific regions of the developing arches is therefore critical to understanding how the facial skeletal bauplan is established.

Genetic studies in a wide range of vertebrates has revealed that patterning of arch NCCs along the dorsoventral axis is an important early step in regionalization of the face, with ventral (distal) cells generating the lower jaw and hyoid bone, maxillary cells forming the upper jaw, and more posteriorly located dorsal (proximal) cells making the lateral upper face. These dorsoventral domains are established in large part by interactions between dorsal Jagged-Notch, ventral/intermediate Endothelin1 (Edn1), and ventral Bmp signaling. Mutation of Edn1 signaling components and key downstream targets (e.g. *Dlx5/6*) in mice and zebrafish results in homeotic transformations and/or losses of skeletal elements derived from the ventral and intermediate domains of the arches, such that the lower jaw adopts an ectopic upper jaw morphology [2–12]. Downregulation of Bmp signaling results in a similar loss of ventral arch-derived structures in mice and zebrafish [13–16], whereas loss of the Notch ligand *jag1b* in zebrafish conversely affects bones and cartilages of the upper/dorsal face, particularly those from the second arch and the dorsal-posterior region of the first arch [17]. These pathways are actively antagonistic: Edn1 and Bmp signaling prevent *jag1b* expression in ventral/intermediate cells, Notch signaling blocks the expression of Edn1 target genes dorsally (e.g. *dlx3b/5a/6a*, *msxe*, *nkx3*.*2*) [17], and Jagged-Notch and Edn1 signaling limit Bmp activity to the most ventral arches in part through upregulation of the Bmp antagonist Gremlin2 in the intermediate domain [13, 14]. The end result of these interactions is the establishment of a distinct dorsal domain (excluding the anterior/maxillary region of the first arch, which is not patterned by Notch [17]) and the subdivision of an initial ventral arch region into distinct ventral/lower and intermediate regions [14]. How this dorsoventral patterning is translated into region-specific cartilage shapes has, however, remained unresolved.

Previous microarray studies of dissected arches in mice lacking the key Edn1 target genes *Dlx5/6* [18] or overexpressing Bmp4 [16] revealed a number of misregulated ventral- and dorsal-specific genes. However, an overarching logic by which the Edn1 and Bmp pathways impart region-specific skeletal shapes remained elusive, with the role of Notch signaling in this process even less clear. In the present study, we perform genome-wide expression analyses of purified arch NCCs to correlate how gene expression patterns change over time in wild-type zebrafish with how gene expression is affected by reduction or elevation of Edn1 or Jagged-Notch signaling. In so doing, we find a prominent role for Jagged-Notch signaling in repressing, and Edn1 in activating, the expression of a set of genes that are strongly induced as arch progenitors mature and begin to acquire cartilage fates, implying that Notch and Edn1 signaling exert opposite effects on cartilage differentiation within the arches.

Two such downstream effectors identified in our genomic analysis are the pre-cartilage condensation marker *barx1* (inhibited by Notch, activated by Edn1) and the early progenitor markers *prrx1a* and *prrx1b* (inhibited by Edn1). In mouse and chick, the homologs of *prrx1a* and *prrx1b* (*Prrx1/PRRX1*, previously called *Prx1* or *mHox*) are expressed in uncondensed preskeletogenic mesenchyme [19–22], whereas *Barx1/BARX1* is found in cells of nascent pre-cartilage condensations that have not or are just beginning to upregulate *Sox9* [23–25]. Studies using a *Prrx1* proximal promoter to drive lacZ expression [22, 26] or Cre recombinase [27] revealed that the cells that make up the limb skeleton and associated connective tissues all pass through a *Prrx1*+ state at some point during their differentiation program. Though no similarly definitive lineage-tracing studies exist for *Barx1*, corollary evidence suggests that most populations of *Barx1*+ cells mature into *Sox9*+ chondrocytes [25, 28–31]. In mammals, PRRX1 is required to repress cartilage differentiation in certain parts of the face: *Prrx1* mouse mutants develop a large ectopic cartilage in place of the dermal squamosal bone on the side of the skull (derived from the dorsal first arch) as well as an aberrant sigmoidal process off of a shortened Meckel’s cartilage; these mutants also show chondrification of the stylohyoid ligament between the styloid process and Reichert’s cartilage (second arch), among numerous other craniofacial and limb skeletal defects [21, 22]. By contrast, impaired cartilage development is observed in *barx1* mutant zebrafish, particularly in the ventral/lower face [32]. In *edn1* mutant zebrafish and mice mutant for the Edn1 receptor (*Ednra2*), defects in ventral and intermediate facial structures are preceded by a loss and shift of *Barx1*/*barx1* expression, particularly in the second arch [10, 33]. These studies indicate that although cartilage differentiation does not strictly require Barx1, chondrogenesis in the ventral and intermediate arches is most sensitive to its loss. Here we demonstrate that early arch patterning pathways compete to drive (Edn1) or restrict (Jagged-Notch, Bmp) the commitment of NCCs to chondrogenic differentiation, in part through antagonistic regulation of *barx1* and *prrx1a/b*. These region-specific differences in the timing and extent of cartilage formation thus establish the template for the later formation of uniquely shaped bones in the upper and lower face.

## Results

### Widespread antagonistic control of arch gene expression by Edn1 and Notch signaling

**Fig 1:**
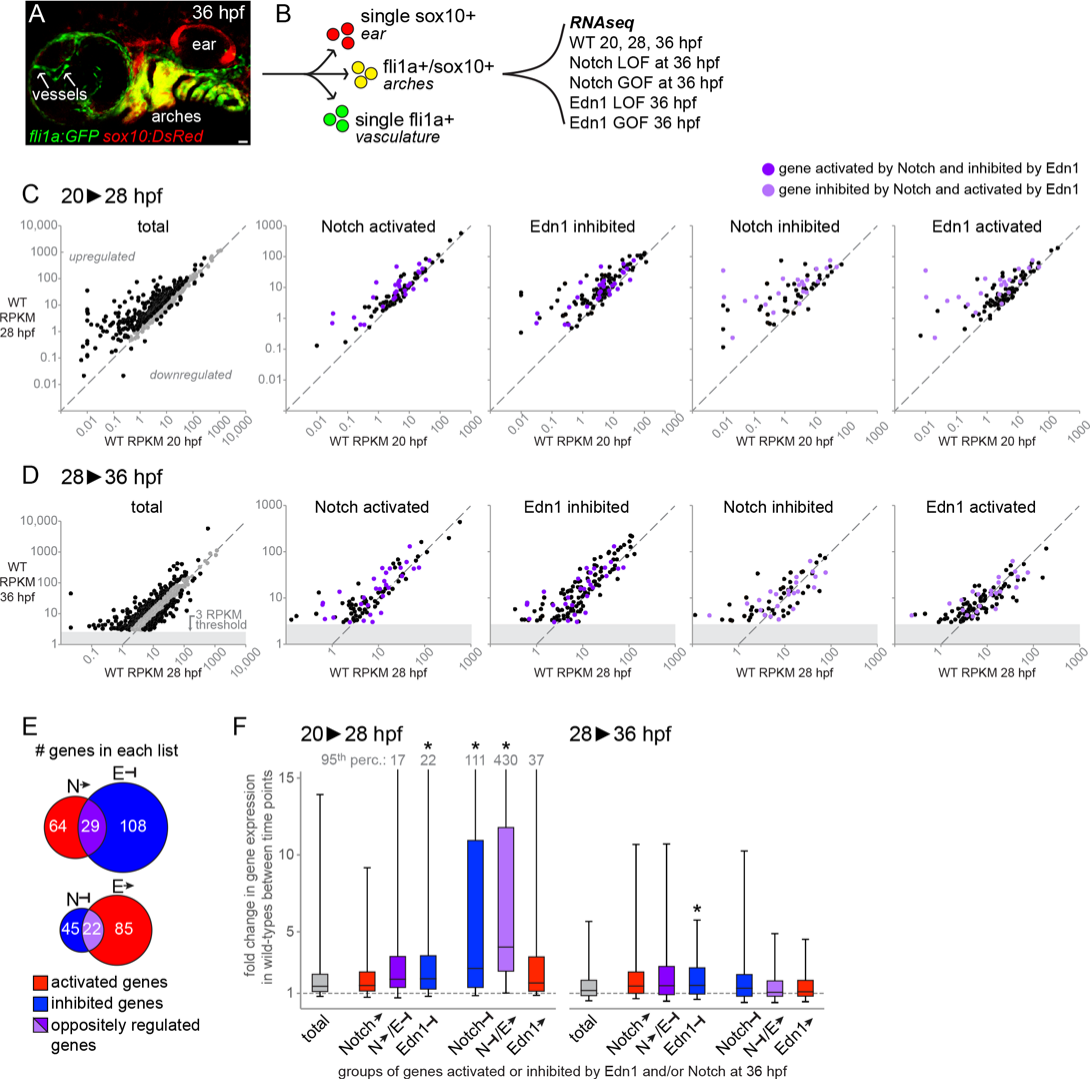
Jagged-Notch signaling represses genes strongly induced during pharyngeal arch differentiation. (A) 36 hpf *fli1a*:*EGFP*; *sox10*:*DsRed* embryo showing colocalization of GFP and DsRed in the arches (yellow), with DsRed-only cells (red) in the ear and GFP-only cells (green) in the vasculature (and macrophages). Scale bar = 20 μm. (B) Double-positive embryos were dissociated and subjected to FACS to isolate GFP/DsRed+ arch cells (yellow) for RNA sequencing. Wild-type embryos were profiled at three stages, and Notch and Edn1 loss- and gain-of-function (LOF, GOF) models at 36 hpf only. (C-D) Scatterplots depicting changes in expression (RPKM) among the genes on each list from 20 to 28 hpf (C) and 28 to 36 hpf (D). Genes to the left of the diagonal were upregulated, while genes to the right were downregulated. For the ‘total’ arch NCC-enriched gene charts, black points indicate genes with fold-changes ≥ 1.5, and grey points indicate genes with fold changes ≤ 1.5. In the other charts, black points correspond to genes uniquely present on a given list, whereas purple points indicate genes oppositely regulated by Notch and Edn1 (dark purple: Notch-activated/Edn1-inhibited; light purple: Notch-inhibited/Edn1-activated). The grey blocks in D reflect the filtering threshold (RPKM ≥ 3 at 36 hpf in wild types) used to generate the total list of arch NCC-enriched genes. (E) Venn diagrams display the numbers of genes on each list, with genes shared between the Notch-activated/Edn1-inhibited or Notch-inhibited/Edn1-activated lists indicated in dark and light purple, respectively. (F) Summary of gene expression changes (RPKM) in wild types between 20 and 28 hpf (left chart) and 28 and 36 hpf (right chart), for genes classified as activated (red) or inhibited (blue) by Notch or Edn1 signaling. The data are shown as the median fold-change value for all genes on a given list (black central line), with flanking second and third quartiles as the lower and upper boxes, respectively, and the 5^th^ and 95^th^ percentiles indicated by the whiskers. For the lists in which the 95^th^ percentile value exceeded the upper boundary of the chart, the value is indicated at the top of the upper whisker. Compared with the total list of arch NCC-enriched genes, genes inhibited by Notch signaling and those that were both inhibited by Notch and activated by Edn1 (light purple) showed a significantly larger increase in expression from 20 to 28 hpf (p < 0.001). Genes inhibited by Edn1 also increased slightly but significantly from 20–28 and 28–36 hpf relative to the total list. The gray dashed line indicates a fold-change value of 1 (no change in expression) between the two time points.

In an unbiased approach towards understanding facial skeletal patterning, we first performed a global gene expression analysis of pharyngeal arch NCCs at three time-points in wild-type embryos. To purify arch NCCs, we conducted fluorescence-activated cell sorting (FACS) on dissociated cells doubly positive for *sox10*:DsRed and *fli1a*:EGFP transgenes (and single-positive and double-negative cells for comparison) (Fig 1A and 1B). *sox10*:DsRed labels all NCCs and the ear, and *fli1a*:EGFP labels arch NCCs, blood vessels, and macrophages. These transgenes uniquely intersect within the arch NCC population, allowing us to selectively enrich for these cells shortly after NCC migration into the arches (20 hours post-fertilization, hpf) and during the initiation of pre-cartilage condensation formation (28 and 36 hpf). cDNA libraries were then constructed for each cell population and subjected to next-generation sequencing. To remove genes with low expression in the arches, we excluded genes with RPKM values ≤ 3 in the wild-type 36 hpf sample. However, a number of genes with known expression in the erthyroid lineage (e.g. hemoglobin genes *hbae1/3*, *hbbe1/3*), macrophages (e.g. *mfap4* [34]), and the ear (e.g. *mvp* [35] and *oc90* [36]) were found in this filtered list, suggesting some degree of contamination of the GFP/DsRed double-positive population by single-positive *fli1a*:*EGFP* or *sox10*:*DsRed* cells. We therefore further filtered for genes with expression ratios of 1.5-fold or higher in the double-positive cells relative to both single-positive populations. This left 536, 668, and 741 arch-enriched genes in the 20, 28, and 36 hpf samples, respectively, with the latter group comprising the “total” arch gene list in Fig 1 (also see S1 Table).

In order to understand how Edn1 and Notch signaling control the expression of these arch NCC-enriched genes, we next performed FACS purification and next-generation cDNA sequencing of GFP/DsRed double-positive cells from 36 hpf embryos with gain or loss of each signaling pathway. Specifically, we compared fold-change differences between *edn1* mutants and stage-matched controls, *jag1b* mutants and wild-type siblings, and *hsp70I*:Gal4; *UAS*:Edn1 or *hsp70I*:Gal4; *UAS*:NICD (Notch1 intracellular domain) versus *hsp70I*:Gal4 controls (subjected to a 20–24 hpf heat-shock to overactivate Edn1 or Notch signaling) (see Methods). The top 20 genes up- and down-regulated in the Edn1 and Notch mutant and overexpression datasets (prior to filtering for arch NCC-enriched genes) are presented in S2 Table. Known targets of Notch (e.g. *jag1b*, *hey2* and *her2/4/15* genes) and Edn1 (e.g. *dlx3b/4a/4b/6a* and *Evf1/2*) are highly represented in these lists. All subsequent analyses were performed using the filtered list of 741 genes with arch-enriched expression in the 36 hpf wild-type sample. To identify those genes most strongly regulated by the Edn1 or Notch pathway, we divided the fold-change of the overexpression (OE) sample by the fold-change of the corresponding mutant (mut) sample. Genes considered ‘activated’ by the Edn1 or Notch pathways had an OE-fold-change/mut-fold-change ratio ≥ 1.5 as well as an OE-RPKM/control-RPKM ratio ≥ 1. Genes considered ‘inhibited’ by Edn1 or Notch had an OE-fold-change/mut-fold-change ratio ≤ 0.667 and a mutant-RPKM/control-RPKM ratio ≥ 1. Lastly, we performed one further refinement for the Notch lists by analyzing genes affected by treatment of embryos with the γ-secretase inhibitor dibenzazepine (DBZ), which blocks processing of the Notch receptor into its active intracellular form [37], starting at 24 hpf. After FACS-purification and next-generation sequencing of double-positive cells from 36 hpf embryos, we calculated the fold-change between DBZ-treated and control samples. Eleven of the top 20 genes downregulated in DBZ-treated embryos belong to the Her/Hes/Hey family of Notch targets [38, 39] (7 of which were shared with the *jag1b* mutant list) (S2 Table), showing that DBZ is primarily affecting Notch signaling in this experiment. However, as γ-secretase inhibitors such as DBZ are also known to affect other signaling pathways [40], we only used the DBZ dataset to further refine the lists generated from the *jag1b* and NICD analyses. Specifically, we excluded genes from the ‘Notch activated’ list that were not also elevated in NICD versus DBZ (fold-change ratio ≥ 1.25) and from the ‘Notch inhibited’ list those not also decreased in NICD versus DBZ (fold-change ratio ≤ 0.8). These filtered gene lists (Fig 1E, S3–S6 Tables) were then used for the global analyses described below.

Consistent with our previous data that the Edn1 and Jagged-Notch signaling pathways antagonize one another during facial development [17], we observed a disproportionately high number of genes oppositely regulated by these pathways. Of the 67 ‘Notch-inhibited’ genes and 107 ‘Edn1-activated’ genes, 22 were in common (Fig 1E). Conversely, 29 genes were in common between the 93 ‘Notch-activated’ and 137 ‘Edn1-inhibited’ genes (Fig 1E). These commonly regulated genes include many known positive Edn1 targets (e.g. *dlx3b*, *dlx4a*, *dlx4b*, *epha4b*, *Evf1/2*, *msxe*, and *notch2*) [5, 17, 18, 33, 41, 42] and negative Edn1 targets (e.g. *jag1b* and *pou3f3a/b*) [17, 18]. Smaller groups of genes were co-regulated by Notch and Edn1 in the same direction (positive, *n* = 9; negative, *n* = 6; S3–S6 Tables), as we previously observed for the BMP antagonist *grem2* [14].

### Jagged-Notch signaling represses genes strongly induced at the onset of facial skeletogenesis

We next examined whether genes activated or inhibited by Notch or Edn1 presented any common temporal signatures during arch development in wild-type embryos. To do so, we first determined the fold changes in wild-type RPKM values for the 741 total arch genes from 20 to 28 hpf and from 28 to 36 hpf (Fig 1C, 1D and 1F; S1 Table). Total arch genes increased by a median of 1.46-fold between 20 and 28 hpf, and 1.18-fold between 28 and 36 hpf. In contrast, we found that the subset of genes that we had annotated as ‘Notch inhibited’ increased 2.62-fold from 20 to 28 hpf in wild types, with many of these upregulated more than 10-fold (p < 0.001). These strongly upregulated genes presented a range of expression levels at 20 hpf, showing that the stronger upregulation of ‘Notch inhibited’ genes is likely not an artifact of these having very low initial expression levels. ‘Edn1 inhibited’ genes also displayed a modest but significantly higher upregulation than total arch genes (median 1.95-fold increase; p < 0.001), though no significant differences were observed for ‘Notch activated’ genes (median = 1.51). Although the ‘Edn1 activated’ genes were not more highly upregulated than total arch genes (median = 1.68), the subset of ‘Edn1 activated’ genes in common with ‘Notch inhibited’ genes were the most strongly induced (median 4.01 fold increase; p < 0.001). Of these 22 common genes, 8 were induced more than 10-fold between 20 and 28 hpf, out of only 48 total >10-fold-upregulated arch genes. At later stages (28–36 hpf; Fig 1D and 1F), only ‘Edn1 inhibited’ genes (median 1.52-fold increase) showed a small but significant difference (p < 0.001) relative to all arch genes (median 1.18-fold increase). In summary, these data show that genes commonly inhibited by Notch and activated by Edn1 are some of the most highly induced during early arch differentiation, consistent with a global role for Notch repression in limiting the differentiation of NCCs in the dorsal arches.

### Accelerated condensation and cartilage formation in the lower face

**Fig 2:**
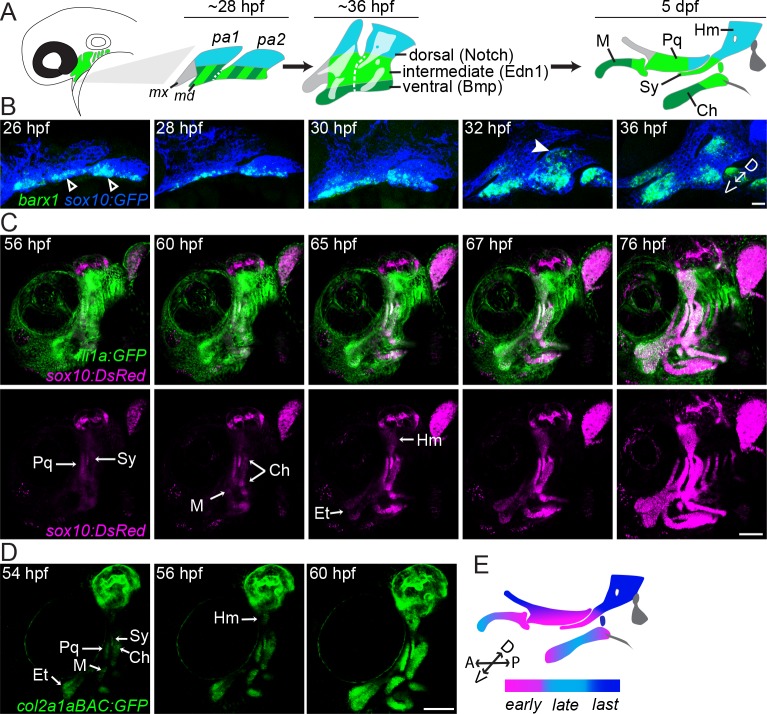
Accelerated cartilage differentiation in ventral-intermediate arch NCCs. (A) Schematic of pharyngeal arch patterning in zebrafish. At early patterning stages (~28 hpf), the first two pharyngeal arches (pa1, pa2) are divided into distinct dorsal (blue) and ventral/intermediate (green stripe) domains, with the latter resolving into intermediate (light green) and ventral (dark green) domains by 36 hpf. Notch activity governs the dorsal domain, Edn1 the intermediate domain, and Bmp signaling the ventral domain. The anterior maxillary domain (grey) is not significantly influenced by any of these pathways. The facial cartilages of the larval skeleton (5 dpf) are color-coded based on their arch origins. Hm, hyomandibula; Pq, palatoquadrate; M, Meckel’s; Sy, symplectic; Ch, ceratohyal. (B) *barx1* (green) is upregulated ventrally (≤ 26 hpf, white open arrowhead) well before dorsal second arch expression can be detected (~32 hpf, white arrowhead). NCCs express the *sox10*:*GFP* transgene (blue). Shown are maximum intensity projections of confocal z-stacks of single-color *in situs* co-stained with a GFP antibody. The orientation of the dorsal (D)-ventral (V) axis is indicated. (C) Stills from a time-lapse movie (see S1 Movie) show the emergence of facial cartilages (*sox10*:*DsRed*+, magenta) from *fli1a*:*EGFP*+ ectomesenchyme (green). *sox10*:*DsRed*+ chondrocytes appear in a stereotyped sequence within the facial cartilages, with cells of the intermediate Sy and Pq cartilages detectable first at 56 hpf, followed by the ventral M and Ch cartilages at 60 hpf and the dorsal Hm at 65 hpf. (D) The same sequence of cartilage differentiation is seen slightly earlier in stills from a time-lapse movie of *col2a1a*_*BAC*_:GFP fish (see S2 Movie). The time-lapses in B and C were performed with a 20x objective using 0.5x digital magnification. Et, ethmoid cartilage. (E) Color-coded schematic of the sequence of chondrocyte differentiation in the facial skeleton. The orientations of the D-V and anterior (A)-posterior (P) axes are indicated. Scale bar in B = 20 μm; scale bars in C, D = 100 μm.

**Fig 3:**
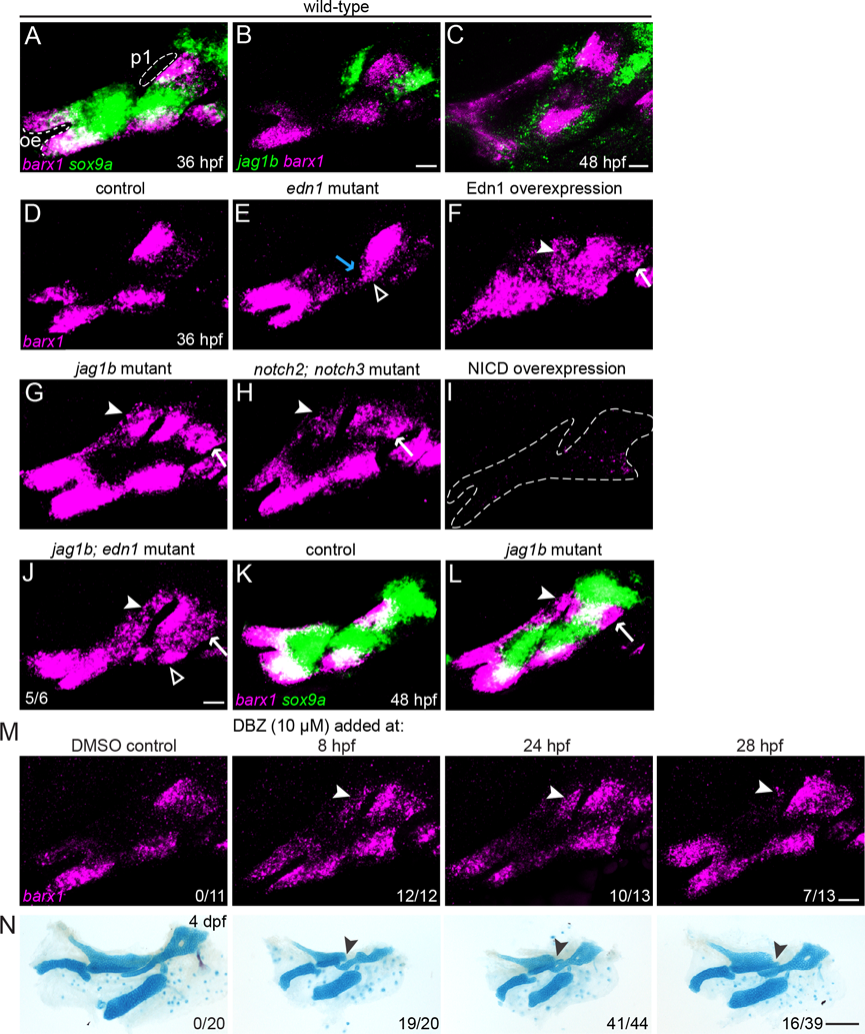
Regulation of *barx1*+ condensations by Edn1 and Notch. (A) At 36 hpf, the intermediate *sox9a* domain (green) only partially overlaps with zones of *barx1* expression (magenta) at the ventral and dorsal poles of each arch. The oral ectoderm (oe) and first pharyngeal pouch (p1) are shown for reference. (B, C) *jag1b* (green) and *barx1* (magenta) are anti-correlated in dorsal NCCs at 36 and 48 hpf. (D-J) *barx1* expression at 36 hpf in the first and second arches of wild-type controls, mutants, and overexpression embryos. Open arrowheads show the loss of ventral *barx1* in *edn1* mutants (E) and its restoration in 5/6 *jag1b*; *edn1* mutants (J). The blue arrow in E indicates weak upregulation of *barx1* in the intermediate domain of *edn1* mutants. Upregulation of *barx1* in the dorsal first arch (white arrowhead) and dorsal second arch (white arrow) is seen in Edn1-overexpressing embryos (F), *jag1b* mutants (G), *notch2*; *notch3* mutants (H), and *jag1b*; *edn1* mutants (J). Dotted lines in (I) show the arches of NICD-overexpression embryos in which *barx1* is nearly absent. (K, L) Ectopic *barx1* persists in *jag1b* mutants at least until 48 hpf, but no ectopic expression of *sox9a* is observed. (M, N) Representative *barx1* expression patterns and skeletal preparations in embryos treated with the Notch inhibitor DBZ (10 μM) starting at the indicated time points. Earlier exposure to the DBZ inhibitor correlated with stronger ectopic *barx1* expression (M) and more severe and penetrant Notch-type skeletal phenotypes (N). Fractions indicate the number of embryos in each treatment that exhibited unambiguous ectopic *barx1* expression in the dorsal first arch (arrowheads in M) or showed posterior Pq malformations (arrowheads in N. DBZ treatment also caused systemic effects, including spinal curvature and cardiac edema, which reduced bone mineralization and led to a general reduction in the size of the craniofacial skeleton. Scale bars in B, C, J, L, M = 20 μm; scale bar in N = 100 μm.

Given the genome-wide role of dorsal Jagged-Notch signaling in repressing strongly induced genes during arch maturation, we investigated whether this might reflect a delay in cartilage differentiation in the dorsal domain versus the rest of the arches. From our genomic analysis, we observed that *barx1*, which marks early pre-cartilage condensations [23, 29], was negatively regulated by Notch signaling (~5-fold lower in NICD versus *jag1b* mutant; S3 Table) and 12.5-fold upregulated between 20 and 28 hpf in wild-type NCCs (S1 Table). By examining a time-course of *barx1* expression (also see [29, 32]), we find *barx1* to be confined to the intermediate/ventral portions of the first and second arches at 26–28 hpf, with maxillary first arch and dorsal second arch expression not initiating until 30–32 hpf (Fig 2B). To determine whether this delay reflects later cartilage differentiation in the dorsal second arch, we made time-lapse recordings of fish expressing *sox10*:DsRed (which shows biphasic expression–first in all NCCs and later in differentiating chondrocytes) along with the arch NCC transgene *fli1a*:EGFP or the chondrocyte transgene *col2a1a*_*BAC*_:GFP (Fig 2C and 2D and S1 and S2 Movies). In both cases, chondrocyte differentiation was first evident in the palatoquadrate cartilage (Pq, primarily an intermediate first arch element with a small amount of dorsal contribution at its posterior end), the symplectic cartilage (Sy, intermediate second arch), at either end of the ceratohyal cartilage (Ch, ventral-intermediate second arch), and the proximal portion of Meckel’s cartilage (M, ventral-intermediate first arch). Chondrocyte transgene expression then spread into the center of the Ch and more ventral portions of the M cartilage. The last elements to differentiate were the hyomandibular cartilage (Hm, dorsal second arch) and the pterygoid process cartilage (Ptp, maxillary) (schematized in Fig 2A and 2E). We also observed that *sox9a*, an early marker of pre-chondrocytes [43–46] that is positively regulated by Edn1 signaling (S6 Table), was expressed only in ventral-intermediate arch NCCs at 36 hpf, with expression spreading to dorsal arch NCCs by 48 hpf (Fig 3A and 3K). Our findings point to cartilage differentiation occurring first in discrete zones, primarily within the intermediate arches, then spreading to other ventral regions and lastly to dorsal regions, consistent with previous studies based on Alcian Blue staining of sulfated proteoglycans typical of cartilage [47].

### Jagged-Notch inhibits and Edn1 promotes *barx1*+ condensations

The earlier chondrogenic differentiation in intermediate/ventral arch cells relative to dorsal cells led us to hypothesize that antagonism between dorsal Jagged-Notch and ventral Edn1 signaling may serve to establish *barx1*+ condensations earlier and/or more extensively in the lower face. As reported previously [10], we find that *barx1* expression is lost from the ventral second but not first arch of *edn1* mutants at 36 hpf (Fig 3E). Conversely, elevation of Edn1 signaling (via 20–24 hpf heat-shock induction of *hsp70I*:*Gal4*; *UAS*:*Edn1* fish) resulted in an expansion of *barx1* expression throughout the arches (Fig 3F). In contrast, we find Jagged-Notch signaling to be required to restrict dorsal *barx1* expression, consistent with our RNAseq data (S3 Table) and the mutually exclusive expression of *barx1* and *jag1b* at 36 and 48 hpf (Fig 3B and 3C). In *jag1b* mutants, *barx1* expression expands into the dorsal-posterior regions of both the first and second arch (Fig 3G; similar to Edn1 overexpression (Fig 3F)), domains that correlate precisely with *jag1b* expression at this stage (Fig 3B). Conversely, *jag1b* expression is unaltered in *barx1* mutants (S1 Fig), indicating that Jagged-Notch signaling functions largely upstream of *barx1* and not vice versa. This ectopic dorsal *barx1* expression was also observed in *notch2*; *notch3* double mutants (Fig 3H), which display similar facial cartilage defects to *jag1b* mutants (consistent with *notch2* and *notch3*, but not *notch1a* or *notch1b*, being expressed in arch NCCs; S2 Fig). Reciprocally, forced activation of Notch signaling in heat-shock-treated *hsp70I*:*Gal4*; *UAS*:*NICD* fish eliminated nearly all *barx1* expression in the arches (Fig 3I). Finally, we find that the positive effect of Edn1 on ventral second arch *barx1* expression can be explained at least in part by the previously reported role of Edn1 in blocking *jag1b* expression [17], as mutation of *jag1b* partially restored ventral second arch *barx1* expression in *edn1* mutants (Fig 3J).

**Fig 4:**
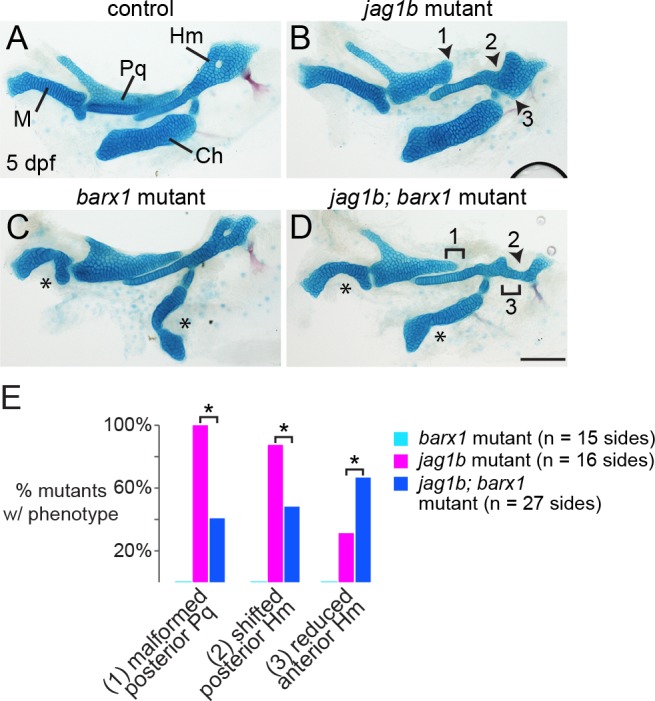
Loss of *barx1* rescues some features of the *jag1b* skeletal phenotype. (A, B) Alcian staining of dissected *jag1b* mutant larval facial cartilages derived from the first and second arches show malformation of the posterior Pq (1), reduction of the anterior Hm (2), and a shift of the posterior Hm (3) such that it abuts the ventral Ch cartilage. (C) In *barx1* mutants, the ventral M and Ch cartilages are reduced (asterisks). (D) In *jag1b*; *barx1* mutants, the Pq truncation (1) and posterior Hm shift (3) are variably rescued, yet loss of the anterior Hm (2) becomes more penetrant. Scale bar = 100 μm. (E) Proportions of mutant larvae exhibiting the indicated phenotypes. None of these skeletal defects were observed in controls or *barx1* mutants. For each phenotype, differences between the genotype groups were significant by Chi-Square test at p < 0.0001 (asterisks).

We next examined whether the ectopic dorsal expression of *barx1* persisted in *jag1b* mutants, as well as the consequences of this for cartilage differentiation. At 36 hpf, *sox9a* expression marks the nascent cartilages in the ventral-intermediate arches that are the first to differentiate, with *barx1* expression in a partially overlapping set of cells nearer to the poles of each arch (Fig 3A). By 48 hpf, *sox9a* expression has spread into the nascent dorsal cartilages yet remains only minimally overlapping with *barx1* (Fig 3K, also see S3 Fig). These results are consistent with previous literature showing that *Barx1* is expressed in nascent pre-cartilage condensations that have not or are just beginning to upregulate *Sox9* [23–25]. In *jag1b* mutants at 48 hpf, we observe an expansion of *barx1* but not *sox9a* expression in the dorsal first and second arch (Fig 3L), suggesting that a subset of dorsal arch NCCs may be trapped in an early *barx1*+ condensation state in the absence of Jagged-Notch signaling. This failure of ectopic dorsal *barx1*+ cells to transition to a more mature *sox9a+* state may help explain why the dorsal cartilages of *jag1b* mutants are truncated rather than expanded (Fig 4B).

Because ectopic dorsal *barx1* expression correlated with dorsal cartilage defects in *jag1b* and *notch2*; *notch3* mutants, we next investigated whether this reflected a common early requirement for Jagged-Notch signaling for both processes. To temporally inhibit Notch signaling, we treated embryos at different stages with 10 μM DBZ, and evaluated the effects on *barx1* expression and skeletal patterning. Although DBZ may also affect other signaling pathways [40], our RNAseq analysis showed that the majority of the most strongly downregulated genes were canonical Notch targets (S2 Table). This analysis focused on the first arch phenotypes, which have proved the most penetrant and consistent across all of our Notch loss-of-function models. Compared with DMSO-treated controls, treatment of embryos with DBZ starting at 8 hpf resulted in a highly penetrant expansion of *barx1* expression into the posterior dorsal first arch (12/12), as well as dorsal cartilage defects similar to *jag1b* mutants (19/20 with Pq malformations; Fig 3M and 3N). DBZ treatment starting at 24 hpf resulted in a weaker and less penetrant *barx1* expansion (10/13 embryos with ectopic first arch *barx1*) and milder dorsal cartilage defects (41/44 with moderate Pq malformations). In contrast, treatment at 28 hpf only mildly affected *barx1* expression in 7/13 embryos, with only 16/39 embryos displaying weak dorsal cartilage malformations (Fig 3M and 3N). Treatments initiated at 32 hpf did not affect *barx1* expression or skeletal patterning. Inhibition of Notch signaling at these stages also had other effects on embryo development, including cardiac edema, which likely contributed to the general reductions in cartilage size. In summary, we observe a tight correlation between *barx1* expression changes and subsequent malformations of dorsal cartilages in Notch-deficient embryos, with the requirement for Notch inhibition by approximately 24 hpf being consistent with the predicted global effects of Notch in repressing arch gene induction between 20–28 hpf (Fig 1F).

### Loss of *barx1* partially rescues the *jag1b* mutant skeletal phenotype

We next investigated the extent to which the ectopic dorsal expression of *barx1* in Notch pathway mutants contributes to the dorsal cartilage malformations. In particular, *jag1b* mutants display several characteristic changes in cartilages of the upper face, including truncation of the posterior end of Pq (i.e. the portion deriving from dorsal first arch NCCs; Fig 2A) and a variable reduction of the anterior part of Hm (Fig 4A and 4B). *jag1b* mutants also display a highly penetrant posterior shift of Hm such that it sits closer to the ventral Ch cartilage [17]. In *barx1* mutants, the dorsal cartilages are largely unaffected, with there instead being conspicuous reductions of the ventral M and Ch cartilages (Fig 4C) [32]. In *jag1b*; *barx1* mutants, we observed an incompletely penetrant rescue of posterior Pq (truncation in 11/27 double mutants versus 16/16 *jag1b* mutants, p < 0.0001) and the position of Hm (posterior shift in 13/27 double mutants versus 14/16 *jag1b* mutants, p < 0.0001) (Fig 4D and 4E). However, ventral M and Ch defects were not restored, and the anterior Hm was more prominently diminished (loss in 18/27 double mutants versus 5/16 *jag1b* mutants, p < 0.0001). Of note, the two regions of skeletal rescue (posterior Pq and posterior Hm) correlate precisely with the earlier ectopic expression of *barx1* in dorsal-posterior first and second arch domains of *jag1b* mutants (Fig 3G), suggesting that the ectopic *barx1* may account in part for these phenotypes. On the other hand, the incompletely penetrant rescue of these elements, in addition to exacerbated phenotypes in other regions (e.g. anterior Hm), indicates the presence of other causative changes in *jag1b* mutants beyond *barx1* misexpression.

### Fate maps reveal Notch dependency for dorsal cartilage growth

**Fig 5:**
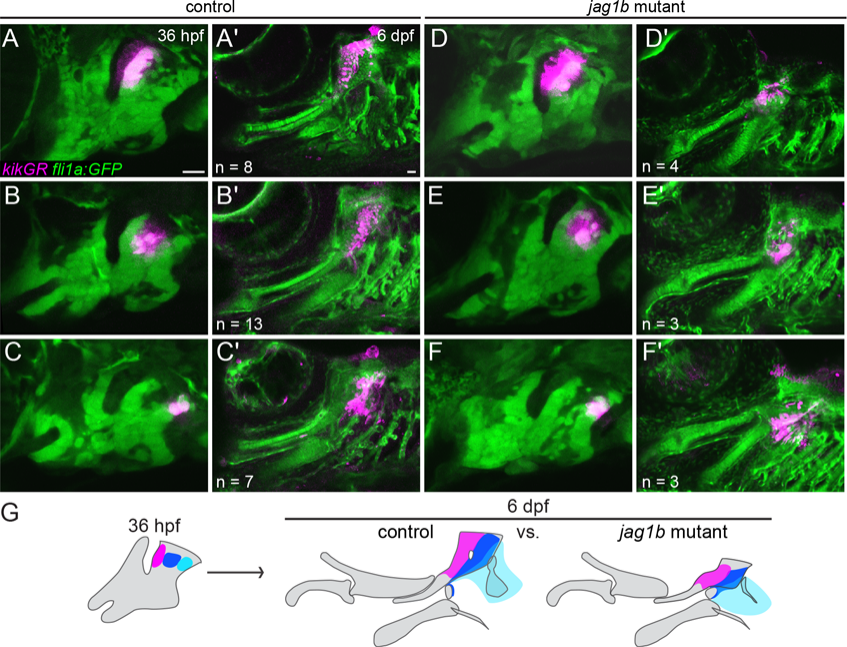
Reduced growth of Hm cartilage in *jag1b* mutants. (A-F) *kikGR* RNA was injected into control *fli1a*:*EGFP* or *jag1b*; *fli1a*:*EGFP* embryos, and kikGR protein was photoconverted in small groups of GFP+ arch NCCs using a UV laser at 36 hpf. The same larvae were then reimaged at 6 dpf to assess contributions of kikGR-converted cells (magenta) to cartilage. In both controls (A-C) and *jag1b* mutants (D-F), photoconverted cells from the anterior, center, and posterior regions of the dorsal second arch contributed to the anterior Hm (A,D), posterior Hm (B,E) and posterior edge of the Hm and opercle bone (C,F). Relative to controls, labeled NCCs from *jag1b* mutants contributed to qualitatively smaller domains of cartilage by 6 dpf. Reproducible differences were seen in each mutant or control examined, with *n* numbers listed in each panel. (G) Summary of these fate maps showing contribution to anterior Hm (red), posterior Hm (dark blue), and opercle bone (light blue). Scale bars = 20 μm.

The finding that posterior-dorsal cells ectopically express *barx1* but fail to turn on *sox9a* in *jag1b* mutants, as well as the fact that cartilage defects were only modestly rescued in *jag1b*; *barx1* double mutants, suggest that Notch signaling has additional roles in dorsal cartilage development. In order to better understand the reductions of dorsal cartilage in *jag1b* mutants, we used photoconversion of the kikGR protein to follow the fate of dorsal second arch NCCs in wild types versus mutants (Fig 5). When wild-type cells were converted at 36 hpf and then re-imaged at 6 days post fertilization (dpf), we found that anterior dorsal second arch NCCs contributed to the anterior portion of the Hm cartilage, central dorsal second arch NCCs to the posterior portion of Hm, and posterior dorsal second arch NCCs to a small amount of Hm and the opercle bone to which it attaches (Fig 5A–5C). In *jag1b* mutants, all three domains contributed to similar portions of the malformed Hm cartilage as in wild types (Fig 5D–5F), indicating no major shift in the fate map of skeletal precursors in mutants. However, whereas cells from all three regions spread along the dorsoventral axis in wild types, cells from comparable domains in *jag1b* mutants gave rise to much smaller domains of cartilage (Fig 5G). These results suggest that Jagged-Notch signaling is also required for the expansion of the dorsal second arch NCCs that generate cartilage in the upper face.

### Edn1 inhibits and Bmp signaling promotes arch expression of *prrx1a* and *prrx1b*

**Fig 6:**
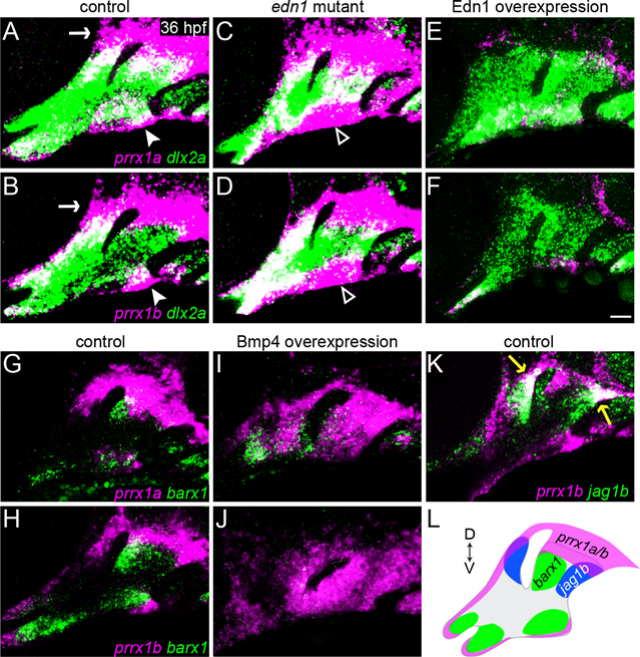
*prrx1a* and *prrx1b* are repressed by Edn1 and activated by Bmp4 signaling. (A-F) Two-color fluorescent *in situs* of 36 hpf wild-type embryos show that, relative to all arch NCCs (*dlx2a*, green), *prrx1a* and *prrx1b* (magenta) are expressed in dorsal arch NCCs and mesenchyme surrounding the ear (white arrow), as well as in a more limited ventral arch domain (white arrowhead). *prrx1a* and *prrx1b* are upregulated in ventral arch NCCs (white open arrowhead) of *edn1* mutants and nearly lost upon overexpression of Edn1 in *hsp70I*:*Gal4*; *UAS*:*Edn1* embryos subjected to a 20–24 hpf heat-shock treatment. (G, H) *prrx1a/b* and *barx1* are expressed complementarily in the arches of wild types. (I, J) Overexpression of Bmp4 in *hsp70I*:*Gal4*; *UAS*:*Bmp4* embryos heat-shocked from 20–24 hpf resulted in broad upregulation of *prrx1a/b* throughout the arches, with *barx1* restricted to domains showing lower *prrx1a/b* expression. (K) *prrx1b* overlaps only slightly with *jag1b* expression at the dorsal-posterior tips of the first and second arches (yellow arrows). (L) Schematic depicting the expression patterns of *prrx1a/b* (magenta), *barx1* (green), and *jag1b* (blue). Scale bar = 20 μm.

While loss of Jagged-Notch signaling can rescue *barx1* expression and ventral cartilage development in *edn1* mutants (Fig 3J; [17]), the partial and largely second-arch nature of this rescue implies the presence of other important pathways downstream of Edn1. Our expression analysis of sorted arch NCCs identified two genes implicated in early skeletogenic mesenchyme identity, *prrx1a* and *prrx1b*, which, like *jag1b*, were upregulated in *edn1* mutants and downregulated in Edn1-overexpressing embryos (S5 Table). Loss of the homologous *Prrx1* gene in mice results in ectopic dorsal facial cartilage [21, 48], implying that Prrx1 genes may also restrict cartilage formation in the upper face. We thus reasoned that Edn1-mediated repression of *prrx1a* and *prrx1b* could help to explain the observed acceleration of cartilage differentiation in the intermediate domain (Fig 2B–2E). Consistently, we observed that expression of *prrx1a* and *prrx1b* was largely excluded from NCCs in the intermediate domain, instead being confined to the dorsal-most and ventral-most poles of the first two arches in 36 hpf wild types (Fig 6A and 6B). However, as predicted by our RNAseq analysis, *prrx1a* and *prrx1b* expression was upregulated along the ventral border and expanded into the intermediate arches of *edn1* mutants, and lost in Edn1-overexpressing embryos (Fig 6C–6F), in accord with the elevated ventral *Prrx1* expression observed in *Dlx5/6* mutant mice [6]. Conversely, overactivation of Bmp4 signaling (via 20–24 hpf heat-shock induction of *hsp70I*:*Gal4*; *UAS*:*Bmp4* fish) upregulated *prrx1a* and *prrx1b* expression throughout the arches (Fig 6G–6J), in accord with previous findings that Bmp signaling promotes genes associated with progenitor status and self-renewal in arch NCCs [16]. Positive regulation by the ventral Bmp signal, combined with negative regulation by intermediate Edn1, could help explain the restriction of *prrx1a/b* expression to the ventral pole of the arches.

We next examined whether the Bmp4 induction of *prrx1a/b* is mediated by Hand2, a strong Bmp target that is specifically expressed in NCCs at the ventral border of the arches in both mice and fish [5, 49], domains that closely overlap with ventral *prrx1a/b* expression. While *Hand2*/*hand2* expression requires positive input from both the Edn1/Dlx and Bmp pathways [5, 6, 13, 49, 50], overexpression of Bmp4 –but not Edn1 –induces its widespread ectopic expression [13, 14], similar to the patterns observed here for *prrx1a/b* (Fig 6G–6J). However, consistent with previous results in mice [51], *prrx1a* and *prrx1b* were expressed largely normally in *hand2* mutants, with a limited expansion of *prrx1a* in the ventral domain (S4 Fig). Thus, Bmp signaling appears to positively regulate *prrx1a/b* expression largely independently of Hand2 function.

### Prrx1 genes are required to repress *barx1* expression and cartilage formation in the dorsal arches

**Fig 7:**
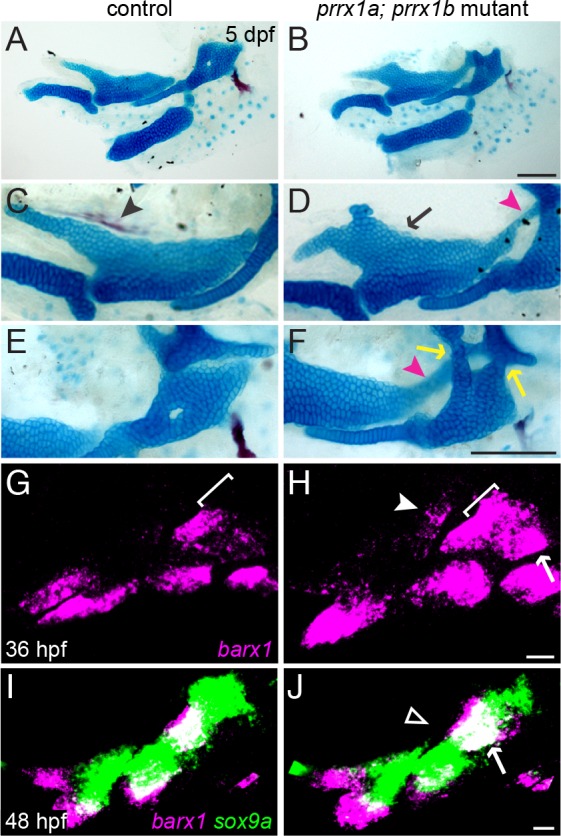
Combined loss of *prrx1a* and *prrx1b* results in ectopic dorsal cartilage. (A-F) *prrx1a*; *prrx1b* mutants develop ectopic cartilage, both from the dorsal-medial surface of Pq (black arrow) and connecting Pq to the otic cartilage (magenta arrowheads), as well as fusions of Hm to the otic cartilage (yellow arrows). The entopterygoid dermal bone (black arrowhead) that normally forms along the dorsal-medial surface of the Pq is also lost. (G, H) In *prrx1a*; *prrx1b* mutants at 36 hpf, *barx1* (magenta) is ectopically upregulated in the dorsal first arch (white arrowhead), along the dorsal border of the second arch (white bracket, compare with G), and in the posterior dorsal second arch (white arrow). (I, J) By 48 hpf in *prrx1a*; *prrx1b* mutants, ectopic *barx1* expression is no longer evident in the posterior first arch (open white arrowhead), and the second arch *barx1*+ domain is slightly larger than the sibling control (white arrow). *sox9a* expression (green) is largely normal at this stage in *prrx1a*; *prrx1b* mutants. Scale bars in B and F = 100 μm; scale bars in H and J = 20 μm.

In order to interrogate Prrx1 function in zebrafish, we used TALENs to generate *prrx1a*^*el558*^ and *prrx1b*^*el491*^ mutant alleles resulting in early truncation of the Prrx1a and Prrx1b proteins upstream of the conserved DNA-binding homeobox domains (S5 Fig). Whereas *prrx1a* and *prrx1b* single mutants did not show craniofacial defects during larval stages (consistent with their identical expression patterns), double homozygous mutants exhibited highly penetrant abnormalities affecting dorsal skeletal elements of the first two arches (Fig 7A–7F). Identical dorsal skeletal phenotypes were seen in double mutants carrying *prrx1a*^*b1246*^ and *prrx1b*^*b1247*^ alleles independently generated by CRISPR-mediated mutagenesis (S5 Fig). In the first arch of double mutant larvae, ectopic cartilage develops along the dorsomedial surface of the Pq cartilage in place of the dermal entopterygoid bone. This extra cartilage is occasionally fused with the trabecular cartilages of the neurocranium. In approximately 40% of double mutant embryos, Pq also extended dorsal-posteriorly to fuse with the otic capsule. In the second arch, the top of the Hm cartilage is malformed, with two highly penetrant cartilaginous fusions to the anterior and middle parts of the otic capsule. The foramen of the Hm, a channel for the VIIth cranial nerve and the anterior lateral line nerve [52], is absent, and the opercle bone is reduced. Despite the expression of *prrx1a/b* at both the dorsal and ventral poles of the arches, double mutants had no detectable defects in ventral cartilages.

Consistent with the ectopic dorsal cartilage, we also found that double mutants displayed ectopic *barx1* expression at earlier stages (36 hpf) in dorsal arch regions that generate the parts of Pq, Hm, and otic cartilages affected in mutants (Fig 7G and 7H). This upregulation of *barx1* in double mutants is consistent with the near mutually exclusive expression of *prrx1a/b* and *barx1* in 36 hpf wild-type embryos (Fig 6G, 6H and 6L). In contrast to *jag1b* mutants (Fig 3L), these ectopic *barx1* expression domains largely disappeared by 48 hpf (Fig 7I and 7J), perhaps accounting for the ectopic formation of cartilage in Prrx1 but not Notch pathway mutants. Interestingly, despite *hand2* being expressed in a similar domain to *prrx1a/b* in the ventral arches, *barx1* has been reported to be lost in *hand2* mutants [32], opposite to the *barx1* expansion we observe in *prrx1a/b* mutants. However, *hand2* expression was unaffected in *prrx1a*; *prrx1b* mutants (S4 Fig), similar to previous observations in *Prrx1*^*-/-*^ mice [53], suggesting that Prrx1 and Hand2 act antagonistically and independently to regulate *barx1* expression and chondrogenesis in the ventral second arch.

### Prrx1a/b and Jagged-Notch signaling function largely independently in the dorsal arches

**Fig 8:**
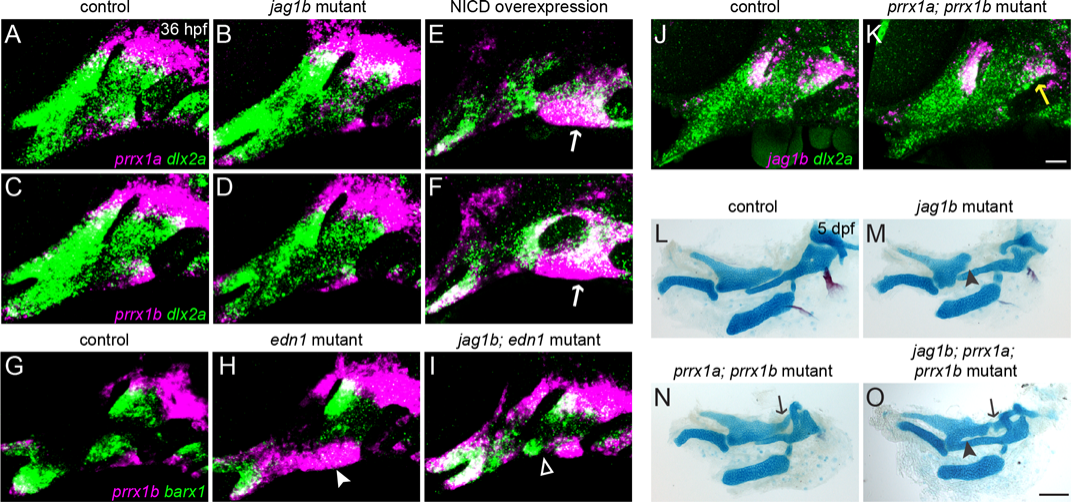
Partially overlapping functions of Prrx1a/b and Jagged-Notch signaling in dorsal cartilage development. (A-F) The expression of *prrx1a* and *prrx1b* (magenta) is largely normal in *jag1b* mutants (B, D) but is upregulated in ventral arch NCCs (white arrows) and reduced in dorsal NCCs upon forced Notch activation in *hsp70I*:*Gal4*; *UAS*:*NICD* embryos subjected to a 20–24 hpf heat-shock treatment. *dlx2a* expression (green) marks all arch NCCs. (G-I) *edn1* mutants display a loss of ventral *barx1* expression (green) and gain of *prrx1b* (magenta) (white arrowhead) (H). In *jag1b*; *edn1* mutants, there is partial recovery of ventral *barx1* expression in the second arch (white open arrowhead), which corresponds to regions where the ectopic expression of *prrx1b* is restored to control levels. (J, K) In 7/10 *prrx1a*; *prrx1b* mutants, *jag1b* expression is partially reduced in the dorsal second arch (yellow arrow). (L-O) Dissections of facial cartilage and bone derived from the first two arches show additive phenotypes in *jag1b*; *prrx1a*; *prrx1b* triple mutants. Similar to *prrx1a*; *prrx1b* double mutants, triple mutants display ectopic cartilage connecting Pq to the otic cartilage (black arrows). However, similar to *jag1b* single mutants, *jag1b*; *prrx1a*; *prrx1b* triple mutants also display irregularities in the main body of Pq (black arrowheads). Scale bar in K = 20 μm; scale bar in O = 100 μm.

Despite both *prrx1a/b* and *jag1b* expression being mutually exclusive to *barx1*, we found only limited overlap between these genes (Fig 6K and 6L). We therefore hypothesized that these pathways function independently to limit *barx1* expression and cartilage formation in distinct domains of the arches. Consistently, we observed no defect in *prrx1a* or *prrx1b* expression in 36 hpf *jag1b* mutants, although forced activation of Notch signaling expanded *prrx1a* and *prrx1b* expression ventrally and decreased it dorsally (Fig 8A–8F). Loss of *jag1b* also partially rescued the ventral expansion of *prrx1b* observed in *edn1* mutants, especially in the second arch (Fig 8G–8I). These findings indicate that, although *jag1b* is not required for *prrx1a/b* expression, high levels of Notch signaling (either artificially or by loss of Edn1) can induce *prrx1b* expression ventrally. Reciprocally, a subset of *prrx1a*; *prrx1b* mutants showed a modest reduction of *jag1b* expression limited to the dorsal posterior second arch (Fig 8J and 8K). To further clarify the genetic interaction between these genes, we analyzed *jag1b*; *prrx1a*; *prrx1b* triple mutants (Fig 8L–8O). In 9/13 triple mutant sides examined, we observed the ectopic posterior extension and fusion of the Pq cartilage to the ear (and not the Pq truncations seen in Notch pathway mutants), indicating that *prrx1a/b* are largely epistatic to *jag1b* with respect to the ectopic Pq phenotype. However, in addition to this extra cartilage, skeletons of the triple mutants (but not *prrx1a*; *prrx1b* double mutants) showed irregular gaps within the body of Pq, reminiscent of abnormalities seen in Notch pathway mutants. These observations support the triple mutant phenotype being largely additive, in line with Prrx1a/b and Jagged-Notch signaling having distinct roles in regulating condensation and cartilage formation in the upper face.

### Simultaneous loss of Prrx1 genes and Jagged-Notch signaling further improves ventral skeletal development in *edn1* mutants

**Fig 9:**
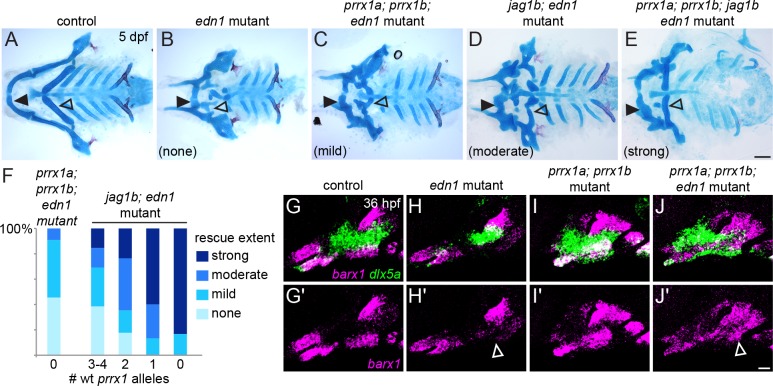
Simultaneous loss of *prrx1a/b* and *jag1b* further improves ventral cartilages in *edn1* mutants. (A-E) Ventral views of dissected facial skeletons. *edn1* mutants have much reduced ventral cartilage in the first (black closed arrowhead) and second (black open arrowhead) arches. Whereas *jag1b*; *edn1* and *prrx1a*; *prrx1b*; *edn1* mutants show some restoration of ventral cartilage, primarily in the second arch, quadruple *prrx1a*; *prrx1b*; *jag1b*; *edn1* mutants show a prominent rescue of second arch Ch cartilage (black open arrowhead) and increased length of first arch M cartilage (black closed arrowhead). (F) Quantification of skeletal rescue in *prrx1a*; *prrx1b*; *edn1* mutants (left column) and *jag1b*; *edn1* mutants with decreasing numbers of wild-type *prrx1a/b* alleles (e.g. 4 wild-type alleles = pure *jag1b*; *edn1* mutants; 0 wild-type alleles = quadruple mutant). Examples of no, mild, moderate, or strong rescue of the ceratohyal cartilage are shown in (B-E). Numbers in each genotype, from left to right: 11, 13, 17, 15, 6. (G-J) *edn1* mutants show a loss of ventral *barx1* expression (magenta, white open arrowhead) and a partial reduction of *dlx5a* (green), which are partially restored in *prrx1a*; *prrx1b*; *edn1* triple mutants. The magenta channel is shown by itself in (G'-J'). Scale bar in E = 100 μm; scale bar in J' = 20 μm.

Because ventral *prrx1a/b* expression increases in the ventral arches of *edn1* mutants (Fig 6C and 6D), we speculated that increased repression of cartilage differentiation by Prrx1 proteins might contribute to the ventral skeletal losses seen in *edn1* mutants. Indeed, we found that homozygous loss of both *prrx1a* and *prrx1b* resulted in a modest rescue of ventral cartilage formation in *edn1* mutants, particularly in the second arch (Fig 9B and 9C), as well as rescue of ventral *barx1* in the second arch and *dlx5a* expression in both the first and second arch (Fig 9G–9J). The partial rescue of ventral cartilage in *prrx1a; prrx1b; edn1* mutants, as well as the earlier recovery of *barx1* and *dlx5a* expression, are qualitatively similar to the phenotypes seen in *jag1b; edn1* mutants (Fig 9D and [17]). By contrast, there was no rescue of ventral *hand2* expression, consistent with our finding that Prrx1a/b do not regulate *hand2* (S4 Fig). As Hand2 normally restricts Dlx expression into the ventral-most arches [54, 55], the lack of *hand2* recovery in the triple mutants may explain the ectopic ventral expansion of *dlx5a* in the *prrx1a; prrx1b; edn1* mutants.

In *jag1b*; *edn1* mutants, the partial recovery of ventral *barx1* expression correlated with zones where *prrx1b* expression was reduced to control levels (Fig 8I). We therefore asked whether the remaining areas of elevated Prrx1 expression in *jag1b*; *edn1* mutants might account for the incomplete rescue. Consistently, we found that progressive reduction of *prrx1a/b* gene dosage in *jag1b*; *edn1* mutants resulted in a progressively better rescue of ventral cartilages, with 5/6 quadruple homozygous *prrx1a*; *prrx1b*; *jag1b*; *edn1* mutants showing a prominent rescue of the ventral second arch-derived Ch cartilage and improved elongation of the first arch-derived M cartilage (Fig 9E and 9F). However, even in these quadruple mutants, the ‘rescued’ ventral cartilages are still smaller than in wild types, and the dorsal skeletal phenotypes associated with *jag1b* and *prrx1a; prrx1b* mutants are still present. These findings reveal important parallel contributions of ectopic Prrx1 and Jagged-Notch activity to the ventral craniofacial defects of *edn1* mutants, yet indicate that Edn1 has additional functions beyond inhibiting Prrx1 and Notch activity.

## Discussion

### Parallel roles of Jagged-Notch and Prrx1 genes in restraining cartilage differentiation in the pharyngeal arches

RNAseq analyses of facial NCCs confirmed our previous findings that Notch acts oppositely to Edn1 during pharyngeal arch development [17]. At a mechanistic level, this global analysis revealed that a major function of Notch signaling is to repress the expression of some of the most strongly upregulated genes in early arch development. These include the homologs of a number of genes implicated in mesenchymal condensation, chondrogenesis, and general skeletogenesis in mammals: e.g. *barx1* [23, 31, 32], *ctgfb* [56], *col6a1* and *col6a6* [57, 58], and *tbx22* [59, 60]. As we only profiled global gene expression patterns in mutants and overexpression embryos at 36 hpf, we cannot conclude whether Notch represses these highly-induced genes only at this later stage, or whether it also restrains their initial upregulation.

The concept of Notch limiting differentiation is becoming a common theme in many developmental and regeneration contexts. For example, sustained Notch signaling in preskeletogenic mesenchyme *in vivo* or mesenchymal progenitors *in vitro* severely abrogates cartilage formation, with cells inappropriately maintained in a precursor state [61–64]. Likewise, Notch signaling promotes regeneration of the caudal fin of zebrafish by maintaining the blastema in a proliferative, undifferentiated state [65, 66]. Though Notch can also promote differentiation in other contexts (e.g. stimulating maturation and hypertrophy in committed chondrocytes [reviewed by [67]]), our findings are consistent with the large body of literature describing roles for Notch in resisting differentiation of progenitor cell populations, in this case specifically in the dorsal arches.

**Fig 10:**
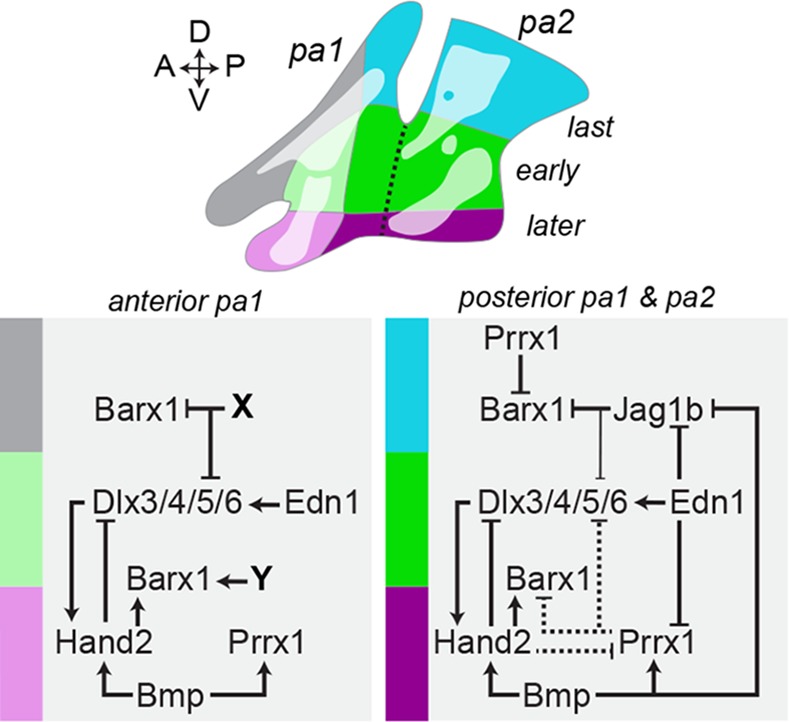
Model of genetic interactions regulating the timing of chondrogenesis in the zebrafish pharyngeal arches. Schematic depicting the approximate origins of the facial cartilages in the early (green), later (purple) and latest (blue and grey) zones of chondrogenesis. In posterior pharyngeal arch 1 (pa1) and pharyngeal arch 2 (pa2), antagonism between Jag1b-Notch and Edn1/Bmp signaling restrains cartilage formation dorsally. Edn1 signaling, possibly acting through Dlx5/6 genes, suppresses dorsal *jag1b* [17] and ventral *prrx1a/b* expression in the intermediate domain (bright green), which allows the earliest initiation of *barx1* expression and chondrogenic differentiation. In the ventral domain (purple), Bmp signaling helps to repress intermediate/ventral *jag1b* expression [13], while persistent expression of *prrx1a/b*–potentially driven by Bmp signaling in a Hand2-independent manner–may help to explain the later onset of chondrogenesis. Hand2 promotes *barx1* [32] while limiting *prrx1a* (but not *prrx1b*) and ventral Dlx expansion [54]. In the dorsal domain (blue), stronger and/or longer repression of *barx1* by combined Prrx1 and Jagged-Notch signaling results in the latest chondrogenic differentiation. Jag1b weakly represses the dorsal expansion of Dlx genes [17]. Prrx1a/b and Jag1b also feed back onto Edn1 signaling to help restrict *dlx5a* expression to the intermediate domain. In anterior pa1, a similar sequence of intermediate to ventral to dorsal/maxillary chondrogenesis is observed, yet Jag1b-Notch and Prrx1 seem to play a less important role, potentially due to different factors (X, Y) restricting *barx1* in the maxillary domain (grey) and promoting *barx1* in the mandibular domain.

Our genomic analysis also identified two *Prrx1* homologs (*prrx1a* and *prrx1b*) as negative targets of Edn1 that function in parallel to Jagged-Notch signaling to restrain cartilage differentiation, yet these pathways appear to do so in different ways (Fig 10). *jag1b* and *prrx1a/b* are expressed in largely non-overlapping domains and are generally not required for the other’s expression. The skeletal phenotypes of Notch and Prrx1 mutants also differ in critical ways. Mutants in both pathways develop ectopic *barx1+* condensations and malformed cartilages in the dorsal arches, but only *prrx1a*; *prrx1b* mutants form ectopic dorsal cartilage. One potential explanation is that ectopic *barx1* expression persists in dorsal NCCs at later stages in *jag1b* but not *prrx1a*; *prrx1b* mutants. Perhaps, Jagged-Notch signaling is also required for ectopic *barx1*+ cells to progress to a *sox9a*+ chondrogenic state. Further, our fate-mapping studies show that dorsal arch NCCs expand less in *jag1b* mutants compared to wild types, which could be due to persistent *barx1* expression restricting the proliferation of chondrogenic cells. However, loss of *barx1* improved only a subset of skeletal defects in *jag1b* mutants, suggesting that skeletal changes in Notch-deficient embryos result from more than just ectopic *barx1* expression. In *prrx1a*; *prrx1b* mutants, the release of dorsal cells from a transient *barx1*+ state may instead allow these cells to reach a critical threshold for making ectopic cartilage (as proposed for the *Prrx1* mouse mutant [68]).

The finding that *prrx1a; prrx1b* double mutants presented skeletal defects only in dorsal elements was somewhat unexpected, given the expression of *prrx1a* and *prrx1b* in both dorsal and ventral arch regions. While homozygous or dominant-negative mutations in *PRRX1* have been associated with loss of the lower jaw in humans [69–73], *Prrx1* mutant mice are similar to zebrafish mutants in displaying ectopic dorsal cartilage, but dissimilar in showing minor abnormalities of the lower jaw [21]; *Prrx1; Prrx2* double mutants display much more pronounced jaw reductions [22, 48, 53]. As zebrafish lack a *Prrx2* homolog [74, 75], the lack of lower jaw defects in *prrx1a; prrx1b* double mutants could reflect redundancy with other pathways, or, alternatively, the evolution of different requirements for Prrx1 genes between fish and mammals. At a molecular level, the ectopic *barx1* expression we observe in the dorsal arches of *prrx1a; prrx1b* mutant fish is reminiscent of the medial expansion of *Barx1* seen in the ventral first arch of *Prrx1; Prrx2* mutant mice [53].

While our data implicate Prrx1 genes and Jagged-Notch as two important negative targets of Edn1 in the ventral arches of zebrafish, the fact that *edn1* mutant phenotypes are only partially rescued by the combined loss of Prrx1a/b and Jag1b suggests other yet to be identified key targets of Edn1. Indeed, our RNAseq analysis revealed two different classes of genes activated by Edn1: (1) those that are highly upregulated during early arch development and also inhibited by Notch (including many well-known Edn1 targets such as *dlx3b/4a/4b/6a*, *hand2*, *epha4b*, *Evf1/2*, and *msxe*) and (2) those that are Notch-independent and only modestly upregulated during early arch development. Given that *jag1b* itself is negatively regulated by Edn1 signaling [17], many of the genes on the first list may in fact be Notch targets that are only indirectly stimulated by Edn1. Functional interrogation of these two classes of target genes should help uncover additional functions of Edn1 in arch development.

Our findings in zebrafish also support a greater role for the Jagged-Notch and Prrx1 pathways in patterning the second arch and posterior half of the first arch compared with the anterior portion of the first arch, which generates the bulk of the lower and upper jaw skeleton (Fig 10). For example, *jag1b* is expressed in only a limited posterior dorsal domain of the first arch and not in the maxillary or mandibular prominences [17], and first arch-derived skeletal structures are less affected than second arch-derived structures in *jag1b* and *prrx1a; prrx1b* mutants. *barx1* expression is also primarily lost in ventral NCCs of the second but not first arch in Edn1 pathway mutants [10, 33], consistent with the more pronounced upregulation of *jag1b* and *prrx1a/b* in this domain. This second arch bias is also reflected by greater rescue of second versus first arch ventral cartilages upon loss of Prrx1 and Notch signaling in *edn1* mutants. Given the very different arrangements of cartilage and bone in the second versus first arch, it is not surprising that programs that restrict cartilage formation have distinct roles in each arch. In the future, it will be interesting to explore how Hoxa2 and Hoxb2, which confer second arch identity [76–79], impact the Notch- and Prrx1-based cartilage restriction programs we have identified.

We also note that other major signaling pathways, such as Bmp, Fgf, Tgfβ, Shh, and Wnt, also influence the spatiotemporal patterns of differentiation within the arches–including control of *prrx1a/b* and *barx1* expression [13, 14, 25, 29, 80–86]. For example, our work indicates that Bmp signaling likely helps to establish *prrx1a/b* expression at the ventral poles of the zebrafish arches. Whether Bmp signaling regulates Prrx1 genes in other vertebrates remains unclear, as previous studies did not detect changes in *Prrx1* expression in conditional *Bmp4* deletion mice [15] or chicken mandibular explants exposed to exogenous Bmp ligands and antagonists [80]. Future work will need to integrate these other key patterning programs into the model to more fully explain how the timing and extent of chondrogenesis is precisely controlled in the developing face.

### Shaping the facial skeleton through temporal control of chondrogenic differentiation

Heterochrony in skeletal differentiation is an important mechanism contributing to the evolution of morphological differences between species [reviewed by [87], also see [88, 89] and references therein]. This concept of variation in developmental timing of homologous structures between species has been proposed to explain, for example, differences in beak length and morphology, as well as the shape of Meckel’s cartilage between quail and duck [90–92]. Our work supports the idea that differential developmental timing can also be a critical driving force for varying skeletal structure within an individual. In the arches of zebrafish, chondrocyte differentiation invariably occurs first in intermediate/ventral before dorsal regions [47]. We have found that these events are presaged by an earlier initiation of *barx1* and *sox9a* expression in intermediate/ventral relative to dorsal arch NCCs, with Jagged-Notch and Prrx1a/b circumscribing the size of the later-forming dorsal condensations.

In a previously proposed ‘hinge-and-caps’ model [93, 94], arch polarity is established by differential signaling in the intermediate regions of the arches (i.e., ‘hinges’) versus the dorsal and ventral poles of the arches (i.e., ‘caps’). Our work provides potential cellular correlates to these hinges and caps in zebrafish, particularly in the second arch and posterior portion of the first arch [95]. We propose that the poles of the arches, or caps, represent progenitor domains, consistent with their expression of the mesenchyme progenitor marker Prrx1 in many species [22, 80, 96]. In contrast, the intermediate arches, or hinges, reflect the sites of initial chondrogenesis, as evidenced by their earlier expression of *sox9a*. Whereas this model predicts that Prrx1 expression at both the ventral and dorsal poles would restrict chondrogenesis relative to the intermediate hinges, dorsal-specific Jagged-Notch signaling would further restrict chondrogenesis in dorsal relative to ventral regions. This model would explain our observations that cartilages generally form first in the intermediate regions, then spread next to the ventral pole, and lastly to the dorsal pole due to combined repressive effects of Prrx1 and Jagged-Notch. However, the timing of cartilage differentiation is clearly more complex. For example, our time-lapse imaging revealed that the Ch cartilage first undergoes chondrogenesis at its tips and then later in its center, potentially correlating with expression of the Bmp target gene *msxe* in a subset of ventral second arch Ch precursors [14]. Hence, layering of additional signaling pathways, such as Bmp, may further refine the timing of cartilage differentiation within the arches.

Given the expression of Prrx1 homologs at the ventral and dorsal poles of the arches from sharks through mammals [96], and conserved expression of *Jag1* in the dorsal arches of mice [97, 98], it appears likely that a similarly regulated intermediate—ventral—dorsal gradient of chondrogenesis may be conserved across vertebrates. For example, in human embryos, Meckel’s cartilage (ventral) differentiates before those elements that form in more proximal/dorsal positions (i.e. the malleus, sphenoid, and styloid process) [99]. On the other hand, differences in the timing and extent of cartilage differentiation might account for the striking differences in facial form between species. In larval zebrafish, the majority of the bony visceral skeleton arises through cartilage templates in the first two arches. In contrast, much of the mammalian facial skeleton forms through direct ossification, with exceptions including Meckel’s cartilage in the lower jaw and the ossicles of the middle ear. These differences might be reflected in the fact that loss of the pre-cartilage marker *Barx1/barx1* has more profound effects on the facial skeleton of zebrafish than mice [32, 100, 101], and, reciprocally, that loss of Prrx1 genes impacts lower jaw development in mammals but not fish [21, 22, 48]. It will therefore be interesting to examine whether differences in the requirements and/or regulation of Prrx1 and Barx1 genes underlie differences in the extent and timing of chondrogenesis between species.

An unanswered question is how heterochrony in cartilage differentiation might translate to the distinct shapes of skeletal elements along the dorsoventral axis. One possibility is that dorsoventral differences in the timing at which progenitors commit to a cartilage fate influences the duration and types of signals they encounter from the surrounding endoderm and ectoderm. For example, Jagged-Notch signaling in the dorsal posterior second arch would protect progenitors from early chondrogenesis, thus allowing these cells to receive later osteogenic cues that direct them to form the large, fan-shaped opercle bone. Such an interpretation is consistent with the reciprocal expansion of *barx1+* pre-cartilage condensations and loss of opercle bone in *jag1b* mutants, and the formation of an ectopic opercle bone upon forced expression of JAG1 in ventral regions [17]. Another possibility is that the timing of condensation formation and subsequent chondrogenesis influences the degree of proliferative expansion of elements in different arch domains [1]. In conclusion, our study revisits heterochrony, a fundamental concept of evolutionary biology, from a developmental perspective, showing that the timing and extent of cartilage differentiation within specific arch regions contributes to the diversity of skeletal shapes within the skull.

## Materials and Methods

### Ethics statement

All zebrafish (*Danio rerio*) were maintained and handled in strict accordance with good animal practices as defined by the relevant national and local animal welfare bodies. Zebrafish embryos were anesthetized for time-lapse imaging or prior to fixation by adding tricaine to their water. All animal experiments performed in this study were approved by the Institutional Animal Care and Use Committee of the University of Southern California (No. 10885, 20193).

### Zebrafish lines

Zebrafish (*Danio rerio*) embryos were reared at 28.5°C and staged as previously described [102]. The following transgenic lines were maintained as heterozygotes: *Tg(fli1a*:*EGFP)*^*y1*^ [103], *Tg(sox10*:*DsRed-Express)*^*el10*^ [104], *Tg(col2a1a*_*BAC*_:*GFP)* [105], *Tg(hsp70I*:*Gal4*)^*kca4/+*^ and *Tg(UAS*:*myc-Notch1a-intra)*^*kca3*^ (hereafter *UAS*:*NICD*) [106], *Tg(UAS*:*Edn1*;*α-crystallin*:*Cerulean)*^*el249*^ and *Tg(UAS*:*Bmp4;cmlc2*:*GFP)*^*el49*^ (hereafter *UAS*:*Edn1* and *UAS*:*Bmp4*, respectively) [14]. The *hsp70I*:*Gal4* and *UAS*:*NICD* lines do not contain selectable markers and were genotyped using primers for Gal4 (F: 5′-CTCCCAAAACCAAAAGGTCTCC-3′; R: 5′-TGAAGCCAATCTATCTGTGACGG-3′) and *UAS*:*NICD* (F: 5’-CATCGCGTCTCAGCCTCAC-3’; R: 5’-CGGAATCGTTTATTGGTGTCG-3’). For the *UAS*:*Edn1* line, in cases where it was not possible to ascertain *α-crystallin*:Cerulean expression in living animals, individuals carrying the transgene were identified by genotyping for the lens marker (F: 5’-TGGTGCAGATGAACTTCAGG-3’ and R: 5’- GCATGCAGACAGCAGCAATA-3’). Gal4 expression was induced in *hsp70I*:*Gal4*; *UAS*:*NICD*, *hsp70I*:*Gal4*; *UAS*:*Edn1*, and *hsp70I*:*Gal4*; *UAS*:*Bmp4* embryos by heat-shocking from 20–24 hpf in a 40°C incubator. The *sucker*/*edn1*^*tf216*^ [5], *jag1b*^*b1105*^ [17], *barx1*^*fh331*^ [32], *notch3*^*fh332*^ [107], and *Df(Chr1)hand2*^*s6*^ [108, 109] mutant lines were described previously and genotyped by PCR using GoTaq (Promega, Madison, WI) with published primer sequences followed by digestion with the appropriate restriction enzymes.

Three new mutant lines (*notch2*^*el515*^, *prrx1a*^*el558*^, *prrx1b*^*el491*^) were generated for this study via TALEN-mediated mutagenesis. The *notch2*^*el515*^ allele was generated with the same TALEN pair used for the previously reported *notch2*^*el517*^ allele [110]. Exon 2 (of 4) of *prrx1a* was targeted with TALENs that recognize the following sequences: Left: 5’-CGTTGAGCTGCTCGTCTGGA-3’; Right: 5’-TGTTTCGCCTCTGTTTACGC-3’, and exon 1 (of 5) of *prrx1b* was targeted with TALENs that recognize the following sequences: Left: 5’-TGGCGAAACGGGCAGGACTA-3’; Right: 5’-TGTATCACTGCCACTCGTTA-3’. TALEN constructs were produced using a PCR-based platform [111]. The TALEN plasmids were linearized by StuI digestion (New England Biolabs, Ipswich, MA), and RNAs were synthesized with the mMessage mMachine T7 Ultra kit (Ambion/Life Technologies, Carlsbad, CA, USA). TALEN RNAs (100 ng/μl) were injected into 1‐cell-stage embryos. Germline founders were identified among the injected individuals by screening outcrossed progeny by PCR followed by restriction digestion. The primers used to identify mutations in each gene are listed in S7 Table. Stable mutant alleles predicted to result in immediate stop codons or frameshifts followed by stop codons were identified by sequencing PCR products in the F1 generation. The *notch2*^*el515*^ allele consists of a 2-bp deletion and a single nucleotide polymorphism (SNP) that destroy a ClaI site in the target region and result in an immediate stop after aa 208 (of 2471), within the extracellular EGF-like domains. The *prrx1a*^*el558*^ allele is an 8-bp deletion that destroys a BseRI site and produces a frameshift after aa 90 (of 245; upstream of the homeodomain at aa 101–155), causing the addition of one incorrect amino acid followed by a stop codon. The *prrx1b*^*el491*^ allele is a 2-bp insertion that abolishes a HinfI site and causes a frameshift after aa 68 (of 245; upstream of the homeodomain at aa 87–165), resulting in the addition of four incorrect amino acids followed by a stop codon. Two additional alleles, *prrx1a*^*b1246*^ and *prrx1b*^*b1247*^, were independently generated via CRISPR-mediated mutagenesis. CRISPR gRNA templates were produced via PCR following a published protocol [112], and gRNAs were synthesized with the MEGAScript T7 transcription kit (Ambion) and column-purified with the mirVana miRNA isolation kit (Ambion). *Cas9* RNA was transcribed from pT3TS-nCas9n with the T3 mMessage kit (Ambion) and purified with an RNeasy Mini Kit (Qiagen, Hilden, Germany) [112]. gRNAs (25 ng/μl) plus *Cas9* RNA (50 ng/μl) were injected into 1‐cell-stage embryos, and stable lines were identified by sequencing as described above. The *prrx1a*^*b1246*^ allele is an 11-bp deletion that causes a frameshift after aa 62, which results in the incorporation of 28 additional amino acids followed by a stop codon. The *prrx1b*^*b1247*^ allele consists of an 8-bp deletion that causes a frameshift after aa 24 and the inclusion of 29 incorrect amino acids before termination. All animal experiments performed in this study were approved by the Institutional Animal Care and Use Committee of the University of Southern California.

### Preparation of FACS-sorted cell populations for RNA sequencing

For RNA sequencing experiments, *fli1a*:*EGFP* fish were crossed to the *sox10*:*DsRed* line, and doubly transgenic *fli1a*:*EGFP*; *sox10*:*DsRed* fish were further crossed to the *edn1*, *jag1b*, *hsp70I*:*Gal4*, *UAS*:*Edn1*, and *UAS*:*NICD* lines. Each of these lines were then separately incrossed to generate embryos for FACS sorting. Wild-type *fli1a*:*EGFP*; *sox10*:*DsRed* (20, 28, and 36 hpf) embryos were sorted for co-expression of GFP and DsRed expression under a fluorescent dissecting stereomicroscope (Leica M165 FC, Wetzlar, Germany) prior to dissociation. Single-positive and double-negative embryos were also saved as controls for FACS. Mutant *edn1*; *fli1a*:*EGFP*; *sox10*:*DsRed* embryos were selected under the fluorescent microscope at approximately 34 hpf based on the reduced distance between the bottom of the first pharyngeal pouch and the ventral border of the arches. To identify *jag1b* mutants and doubly-transgenic *hsp70I*:*Gal4*; *UAS*:*Edn1* or *hsp70I*:*Gal4*; *UAS*:*NICD* individuals, we genotyped cell lysates of tail biopsies collected from anesthetized individual 24-hpf *fli1a*:*EGFP*; *sox10*:*DsRed* double-positive embryos. To induce Edn1 or NICD overexpression in the *hsp70I*:*Gal4*; *UAS*:*Edn1* and *hsp70I*:*Gal4*; *UAS*:*NICD* lines, embryos were heat-shocked from 20–24 hpf in an incubator set at 40°C. As another means of inhibiting Notch signaling, *fli1a*:*EGFP*; *sox10*:*DsRed* embryos were treated with dibenzazepine (DBZ; Tocris, Bristol, UK; final concentration of 10 μM in embryo medium) from 24–36 hpf. The number of embryos used for each sort and the number of cells obtained are presented in S9 Table.

To facilitate FACS analyses at the 36 hpf time point, embryos were moved at 27 hpf to an incubator set at 22°C to delay their development such that they reached an approximation of the 36 hpf stage the following morning. *fli1a*:*EGFP*; *sox10*:*DsRed* double-positive embryos were dissociated following [113], with minor modifications. Briefly, 30–40 dechorionated embryos were incubated in fresh Ringer’s solution for 5–10 minutes and agitated by pipetting to remove the yolk. The deyolked embryos were then mixed with a protease solution containing 0.25% trypsin (Life Technologies), 1 mM EDTA, and 2 mg/ml Collagenase P (Roche Life Science, Indianapolis, IN) in PBS and incubated at 28.5°C for 15 min, pipetting up and down every 5 min to aid the dissociation. The reaction was stopped by the addition of a 6x stop solution consisting of 6 mM CaCl_2_ and 30% fetal bovine serum (FBS) in PBS. The cells were pelleted via centrifugation at 2000 rpm for 5 min at 4°C, resuspended in suspension medium (1% FBS, 0.8 mM CaCl_2_, 50 U/ml penicillin, and 0.05 mg/ml streptomycin (Sigma-Aldrich, St. Louis, MO) in phenol red-free Leibovitz’s L15 medium (Life Technologies)), pelleted again as above, and then resuspended in 500 μl suspension medium and placed on ice. Cells were sorted by FACS for GFP and DsRed expression on a MoFlo Astrios instrument (Beckman-Coulter, Brea, CA, USA). GFP/DsRed double-positive, double-negative, and single-positive populations were collected directly into RLT lysis buffer (Qiagen). Total RNA was immediately extracted using the RNeasy Micro kit (Qiagen) following the manufacturer’s protocol and quantified on a NanoDrop 2000 spectrophotometer (NanoDrop Products, Wilmington, DE, USA).

### cDNA library preparation and RNA sequencing

The quality and quantity of extracted RNA were assessed on a Bioanalyzer Pico RNA chip (Agilent, Santa Clara, CA). cDNA was then made from the extracted RNA using the SMARTer V3 kit (Clontech, Mountain View, CA), according to the manufacturer’s instructions. The number of amplification cycles for cDNA synthesis was estimated based on input amounts of RNA. The size and amount of the resulting cDNA were then confirmed by Bioanalyzer. Sonication was performed on a S2 ultrasonicator (Covaris, Woburn, MA) according to Clontech’s recommended conditions. DNA libraries were constructed using the Kapa Hyper prep kit (Kapa Biosystems, Wilmington, MA) and NextFlex adapters (Bioo Scientific, Austin, TX). Libraries were visualized by Bioanalyzer analysis and quantified by qPCR (Kapa library quantification kit). Sequencing was performed on Illumina HiSeq 2000 (50-bp paired end reads) and NextSeq 500 (75-bp paired end reads) machines (Illumina, San Diego, CA). DNA libraries were constructed and sequencing was performed at the Norris Cancer Center Molecular Genomics Next Gen Sequencing Core at USC.

### Sequencing data analysis

Raw sequencing data in Fastq format was imported into the Partek Flow interface for alignment and quantification. Pre-alignment QC showed that the reads from all samples had generally high quality, with the average Phred quality score for each sample being above 30. Reads were then trimmed from both ends based on Phred quality score with a minimum end quality level of 20 and a minimum acceptable read length of 25. The TopHat 2 algorithm was used to align the trimmed reads to the zebrafish GRCz10 genome assembly (Ensembl_v80). Aligned reads were then quantified using the Partek E/M algorithm with default parameters to yield the RPKM values. RNAseq files have been deposited in NCBI’s Gene Expression Omnibus and are accessible through the GEO Series accession number GSE72985. Filtered gene lists were derived in MS Excel as described in the Results section. Six genes on the list of arch NCC-enriched genes had passed the ≥ 3 RPKM threshold at 36 hpf but had RPKM values of 0 in the 20 hpf sample, leading to a division error that would have precluded their inclusion in the temporal expression analysis; we thus set the 20 hpf RPKM value for these genes to 0.01 based on the lowest positive RPKM values in the dataset.

### Skeletal analysis and *in situ* hybridization

Alcian Blue and Alizarin Red staining to detect cartilage and bone, respectively, was performed on 4–6 dpf larvae as previously described [114]. Two-color fluorescent *in situ* hybridizations were carried out as previously reported [17]. Published probes used in this study include *dlx2a* [115], *dlx5a* [10], *notch2*, *jag1b* [17], and *sox9a* [44]. Partial cDNAs for *barx1*, *notch1a*, *notch1b*, *notch3*, *prrx1a*, and *prrx1b* were cloned into the pCR-Blunt II-TOPO vector (Life Technologies) and sequence-verified prior to plasmid linearization and *in vitro* transcription with Sp6 or T7 polymerase (Roche) (S8 Table).

### Notch inhibitor treatments

To determine when Notch signaling affects skeletal patterning, we treated embryos with the γ-secretase inhibitor DBZ. DBZ dissolved in dimethyl sulfoxide (DMSO; 10 mM stock) was added to embryo medium to a final concentration of 10 μM. Embryos (*n* = 30–50 per treatment) were incubated in this solution starting at 8, 24, or 28 hpf until fixation at 36 (8 hpf group) or 42 hpf (other groups) for *in situs* or at 4 dpf for Alcian and Alizarin staining. In the groups used for skeletal staining, the DBZ solution was refreshed at 48 hpf and thoroughly washed out at 56 hpf. Clutch-mate controls were exposed to the same concentration of DMSO. Embryos were dechorionated at 24 hpf to improve drug accessibility.

### Fate maps

Fate-mapping was performed with the green-to-red photoconvertible kikGR protein [116]. *kikGR* RNA was injected into embryos from a *jag1b*; *fli1a*:*EGFP* cross at the one-cell stage. At 36 hpf, embryos were anesthetized in Tricaine (Sigma-Aldrich) and mounted in 0.2% agarose for confocal imaging. Small groups of cells in the dorsal arches were selected using the region of interest tool in the Zeiss LSM software and exposed to the UV 405 laser until the red photoconverted protein became apparent (typically ~10 seconds using 50% laser power). The same animals were reimaged at 6 dpf to determine the destination of the photoconverted cells and then genotyped for the *jag1b*^*b1105*^ mutation.

### Imaging

Confocal images of in situ hybridizations (~30 μM z-stacks) were captured on a Zeiss LSM5 microscope using ZEN software. Time-lapse imaging of doubly transgenic *fli1a*:*EGFP*; *sox10*:*DsRed* and *col2a1a*_*BAC*_:*GFP*; *sox10*:*DsRed* larvae followed [117], with ~130 μM of z-stacks collected every 10 or 12 minutes starting at 48 hpf. Skeletal preparations were photographed using a Leica DM2500 microscope. Image levels were adjusted in Adobe Photoshop CS6, with care taken to apply identical adjustments to images from the same data set and to avoid removing information from the image.

### Data analysis

To analyze changes in gene expression between 20 and 28 hpf and 28 and 36 hpf for the total arch and Edn1- and Notch-regulated gene lists, we first calculated the median and quartile values for each list. The full lists were then collectively compared first by a Kruskal-Wallis test and then pairwise by Mann-Whitney U tests, with the Bonferroni correction applied to an α-value of 0.05 to account for multiple comparisons. Chi-square was used to compare the proportions of embryos showing different skeletal phenotypes in *jag1b*, *barx1*, and *jag1b*; *barx1* mutants, with p < 0.05 considered significant. JMP 7.0 software (SAS) was used for statistical analysis. Numbers for each experiment are presented in S10 Table.

## Supporting Information

S1 Table Total arch-enriched genes. (XLSX)

S2 Table Top 20 genes downregulated and upregulated in each loss and gain-of-function model. (XLSX)

S3 Table Notch-inhibited genes. (XLSX)

S4 Table Notch-activated genes. (XLSX)

S5 Table Edn1-inhibited genes. (XLSX)

S6 Table Edn1-activated genes. (XLSX)

S7 Table Genotyping primers used for new TALEN alleles. (XLSX)

S8 Table *In situ* probe construction. (XLSX)

S9 Table Summary of FACS analysis for RNAseq. (XLSX)

S10 Table Experimental numbers. (XLSX)

S1 Fig *jag1b* expression in *barx1* mutants. *jag1b* expression (magenta) in posterior-dorsal cells of the first and second arches is indistinguishable between controls (A) and *barx1* mutants (B) at 36 hpf. *dlx2a* (green) marks all arch NCCs. Maximum intensity projections of confocal z-stacks. Scale bar = 20 μm.
(TIF)

S2 Fig Combined mutation of *notch2* and *notch3* causes dorsal skeletal defects. (A-D) Expression of Notch receptors in the pharyngeal arches. At 36 hpf, *notch1a* (A, magenta) and *notch1b* (B, magenta) are expressed in the ectodermal cleft adjacent to the first pharyngeal pouch but are undetectable in *dlx2a*+ NCCs (green). By contrast, *notch2* (C) is strongly expressed in intermediate/dorsal NCCs (white arrow), and *notch3* is expressed in dorsal second arch NCCs (white arrow), arch core mesoderm (*dlx2a*-negative, orange arrow), and the first ectodermal cleft (white arrowhead). (E-I) Mutation of *notch2* (F) or *notch3* (G) alone does not affect skeletal patterning, though a subset of *notch2*^*-/-*^; *notch3*^*+/-*^ mutants (H) show some *jag1b*-like dorsal skeletal defects. Combined loss of both genes (I) results in a severe phenotype (two examples shown in I, I') consisting of a significant reduction in the size of the Hm and a shift towards a more posterior position (black open arrowhead), variable fusions of the second arch joint (black arrow), and abnormalities in the posterior Pq (black arrowhead). The overall size reduction and failure of bone mineralization are likely non-specific consequences of cardiac edema. Scale bar in D = 20 μm; scale bar in I = 100 μm.
(TIF)

S3 Fig *barx1* and *sox9a* mark different states of skeletal differentiation. Individual confocal sections of a *barx1* (magenta) and *sox9a* (green) *in situ* hybridization in a wild-type embryo at 48 hpf, showing that the two markers are largely mutually exclusive, with the exception of the ventral tip of the forming Meckel’s and ceratohyal cartilages (white arrowheads in z12). Scale bars in all panels = 20 μm.
(TIF)

S4 Fig Cross-regulation of *prrx1a/b* and *hand2* expression. In *hand2* mutants, expression of *prrx1a* (A, B; magenta) is slightly upregulated (arrowhead) in ventral cells, and *prrx1b* (C, D; magenta) is unaltered. (E, F) *hand2* expression (magenta) is unaffected in *prrx1a; prrx1b* mutants. *dlx2a* (green) marks all arch NCCs. (G-J) *hand2* expression (magenta) is not rescued in *prrx1a; prrx1b; edn1* triple mutants (J) compared with *edn1* single mutants (I). Dashed lines indicate approximate arch boundaries. Scale bar = 20 μm.
(TIF)

S5 Fig Independently derived *prrx1a*; *prrx1b* mutants confirm specificity of skeletal phenotype. (A, B) Independently derived *prrx1a*^*b1246*^; *prrx1b*^*b1247*^ double mutants (B, B') phenocopy *prrx1a*^*el558*^; *prrx1b*^*el491*^ mutants (Fig 7B). Note the expanded cartilage in the palatoquadrate (black arrowhead) and the fusions between the hyomandibula and otic cartilage (black arrow). Scale bar = 100 μm. (C) Schematic of *prrx1a/b* TALEN (el558, el491) and CRISPR (b1246, b1247) alleles. In all cases, the mutant allele produces a frameshift (black) and premature stop codon upstream of the homeobox (Hx) and OAR domains (O).
(TIF)

S1 Movie Delayed chondrocyte differentiation in the dorsal second arch. Time-lapse movie of a *fli1a*:*EGFP*; *sox10*:*DsRed* embryo showing the invariant sequence of emergence of differentiating chondrocytes (*sox10*:*DsRed*+, magenta) from arch ectomesenchyme (*fli1a*:*EGFP+*, green) first in intermediate and then ventral elements and finally in the dorsal hyomandibula. Imaging of the 125-μM z-stack was initiated at 48 hpf and continued for 24 hpf. Stills from this movie are presented in Fig 2B.
(MP4)

S2 Movie Two chondrocyte markers are upregulated across the developing face in the same sequence. Time-lapse movie of a *col2a1a*_*BAC*_:*GFP*; *sox10*:*DsRed* embryo showing that, similar to *sox10*:*DsRed* (magenta), a GFP transgene (green) driven by regulatory information in the *col2a1a* genomic locus is also upregulated in intermediate/ventral cartilages prior to the dorsal hyomandibula, though GFP expression can be detected several hours before DsRed becomes apparent, likely due to transgene effects. Imaging of the 150-μM z-stack was initiated at 48 hpf and continued for 24 hpf. Stills from this movie are presented in Fig 2C.
(MP4)
