# Supplementary figures and images for: Competition between Jagged-Notch and Endothelin1 Signaling Selectively Restricts Cartilage Formation in the Zebrafish Upper Face
Source: PLoS Genet. 2016 Apr 8;12(4):e1005967. doi: 10.1371/journal.pgen.1005967 (PMC4825933; doi:10.1371/journal.pgen.1005967)

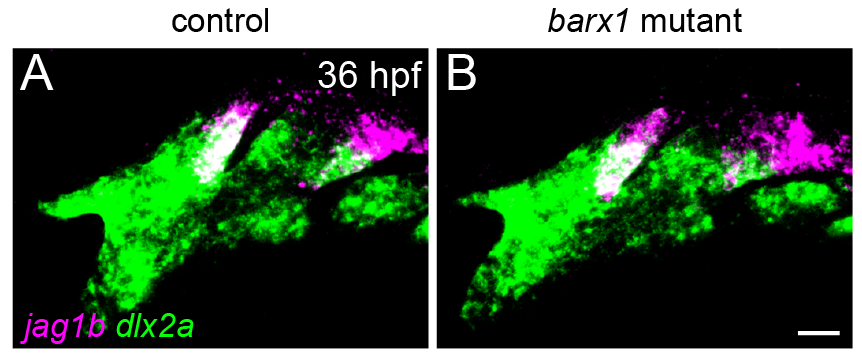

Supplement: S1 Fig — jag1b expression (magenta) in posterior-dorsal cells of the first and second arches is indistinguishable between controls (A) and barx1 mutants (B) at 36 hpf. dlx2a (green) marks all arch NCCs. Maximum intensity projections of confocal z-stacks. Scale bar = 20 μm. (TIF) [file pgen.1005967.s011.tif]

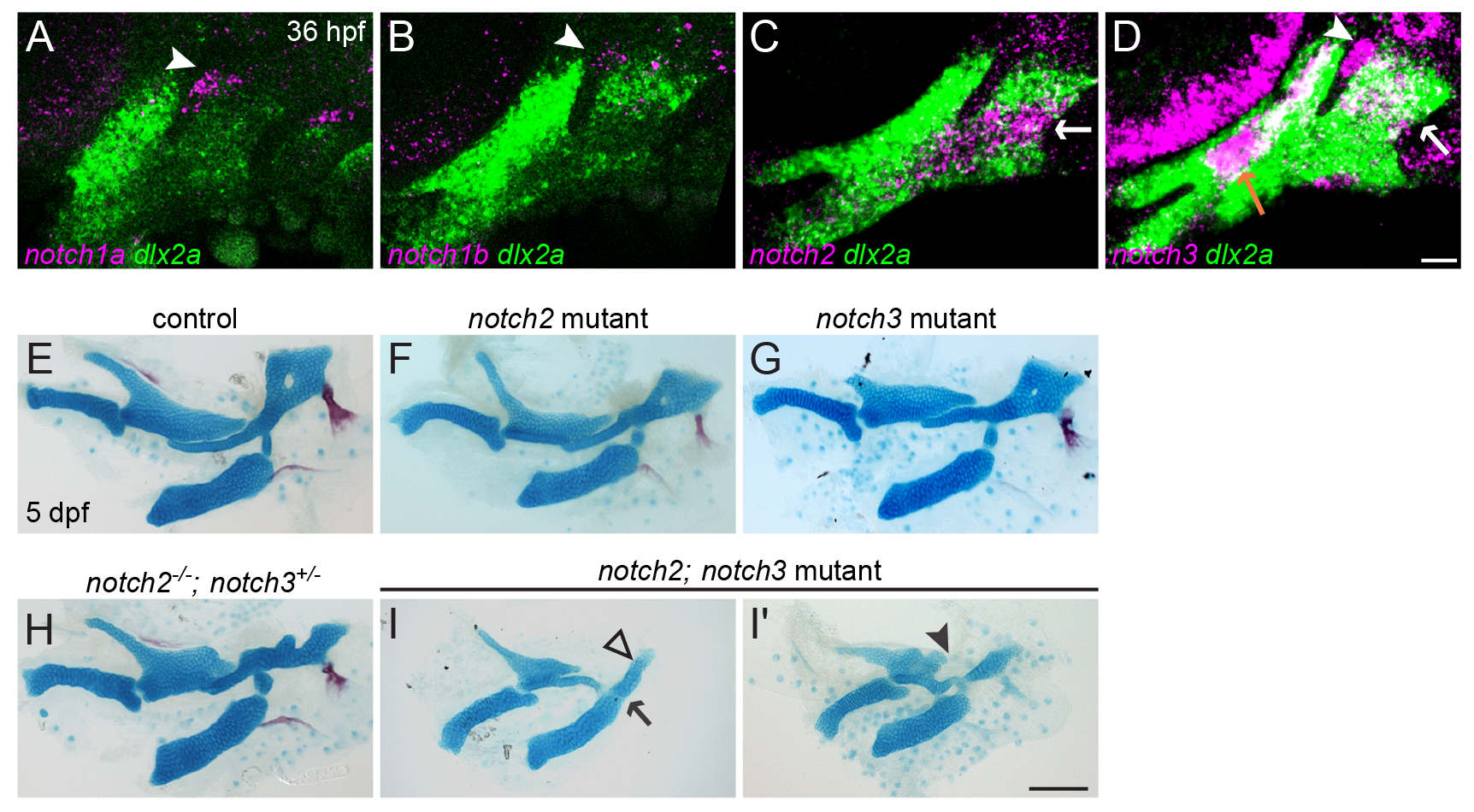

Supplement: S2 Fig — (A-D) Expression of Notch receptors in the pharyngeal arches. At 36 hpf, notch1a (A, magenta) and notch1b (B, magenta) are expressed in the ectodermal cleft adjacent to the first pharyngeal pouch but are undetectable in dlx2a+ NCCs (green). By contrast, notch2 (C) is strongly expressed in intermediate/dorsal NCCs (white arrow), and notch3 is expressed in dorsal second arch NCCs (white arrow), arch core mesoderm (dlx2a-negative, orange arrow), and the first ectodermal cleft (white arrowhead). (E-I) Mutation of notch2 (F) or notch3 (G) alone does not affect skeletal patterning, though a subset of notch2-/-; notch3+/- mutants (H) show some jag1b-like dorsal skeletal defects. Combined loss of both genes (I) results in a severe phenotype (two examples shown in I, I') consisting of a significant reduction in the size of the Hm and a shift towards a more posterior position (black open arrowhead), variable fusions of the second arch joint (black arrow), and abnormalities in the posterior Pq (black arrowhead). The overall size reduction and failure of bone mineralization are likely non-specific consequences of cardiac edema. Scale bar in D = 20 μm; scale bar in I = 100 μm. (TIF) [file pgen.1005967.s012.tif]

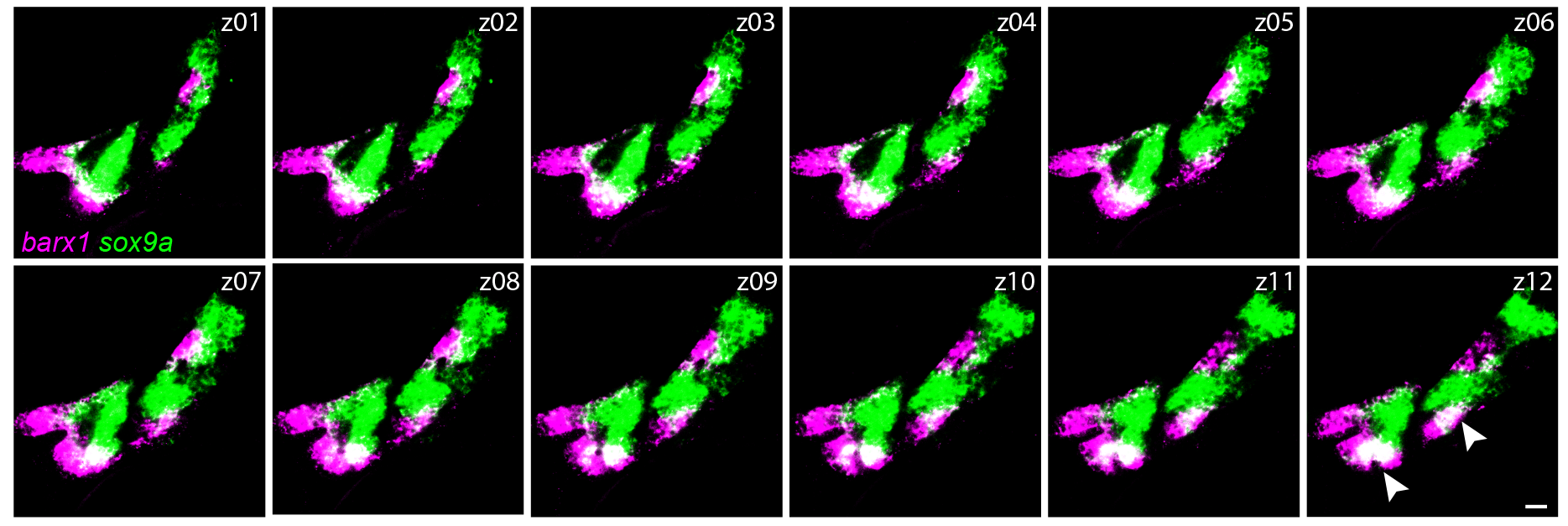

Supplement: S3 Fig — Individual confocal sections of a barx1 (magenta) and sox9a (green) in situ hybridization in a wild-type embryo at 48 hpf, showing that the two markers are largely mutually exclusive, with the exception of the ventral tip of the forming Meckel’s and ceratohyal cartilages (white arrowheads in z12). Scale bars in all panels = 20 μm. (TIF) [file pgen.1005967.s013.tif]

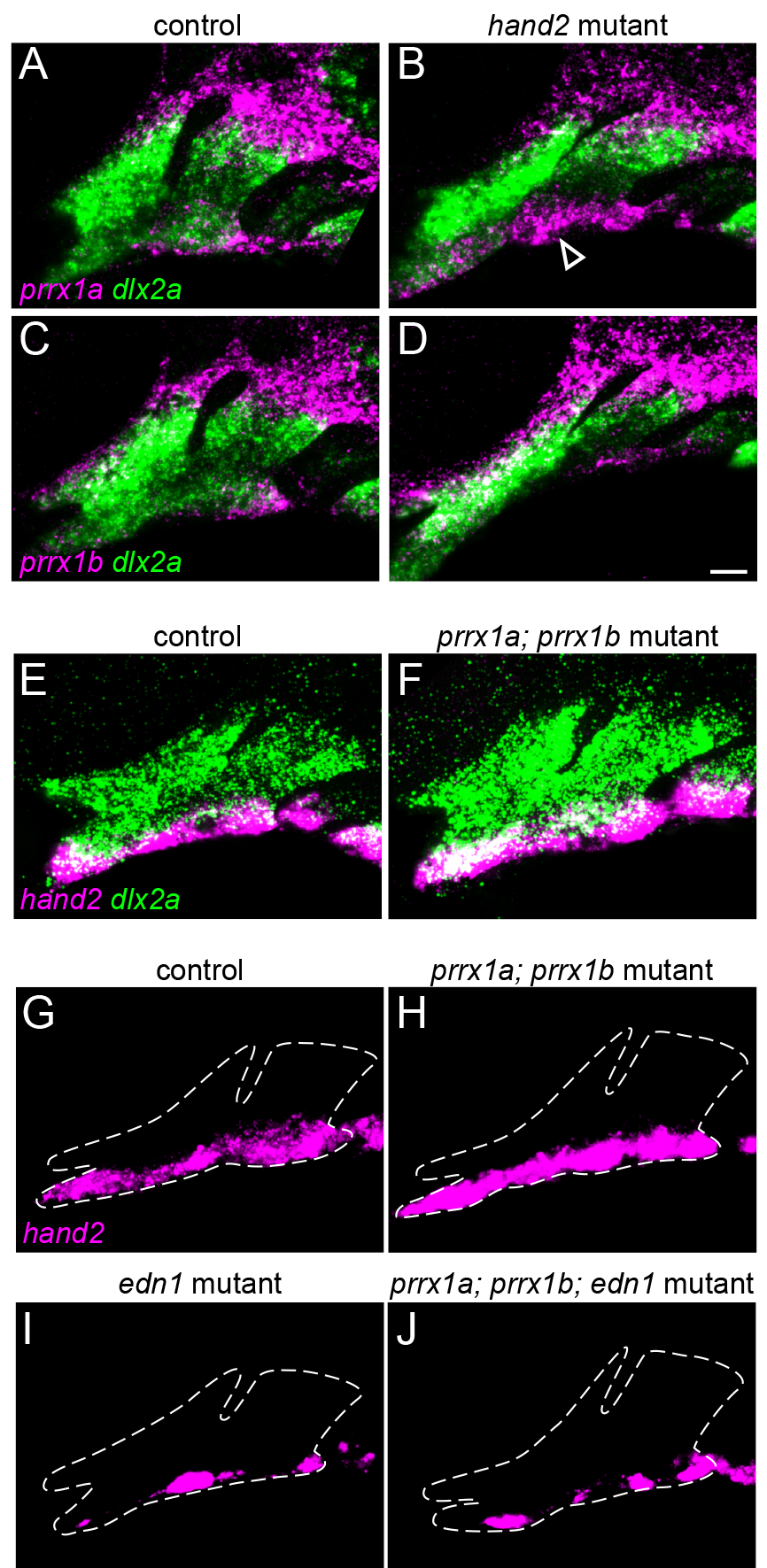

Supplement: S4 Fig — In hand2 mutants, expression of prrx1a (A, B; magenta) is slightly upregulated (arrowhead) in ventral cells, and prrx1b (C, D; magenta) is unaltered. (E, F) hand2 expression (magenta) is unaffected in prrx1a; prrx1b mutants. dlx2a (green) marks all arch NCCs. (G-J) hand2 expression (magenta) is not rescued in prrx1a; prrx1b; edn1 triple mutants (J) compared with edn1 single mutants (I). Dashed lines indicate approximate arch boundaries. Scale bar = 20 μm. (TIF) [file pgen.1005967.s014.tif]

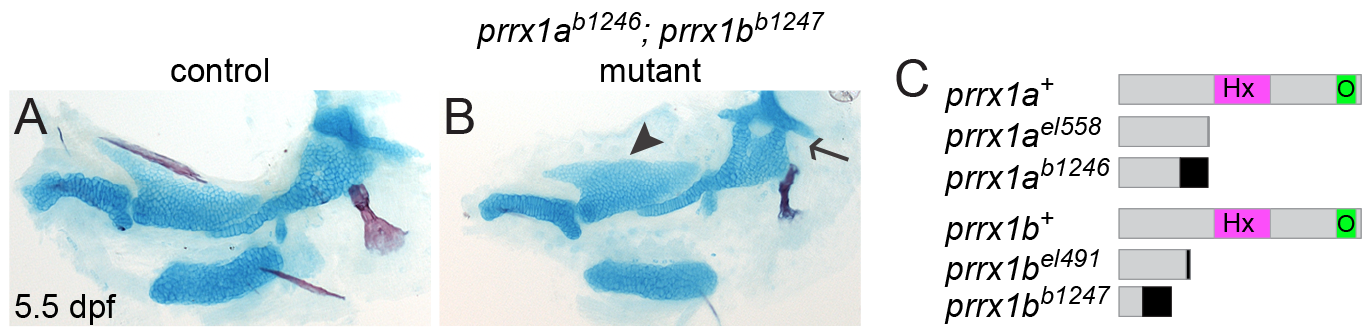

Supplement: S5 Fig — (A, B) Independently derived prrx1ab1246; prrx1bb1247 double mutants (B, B') phenocopy prrx1ael558; prrx1bel491 mutants (Fig 7B). Note the expanded cartilage in the palatoquadrate (black arrowhead) and the fusions between the hyomandibula and otic cartilage (black arrow). Scale bar = 100 μm. (C) Schematic of prrx1a/b TALEN (el558, el491) and CRISPR (b1246, b1247) alleles. In all cases, the mutant allele produces a frameshift (black) and premature stop codon upstream of the homeobox (Hx) and OAR domains (O). (TIF) [file pgen.1005967.s015.tif]
